# Entropy removal of clinical features

**DOI:** 10.1038/s41598-025-29069-0

**Published:** 2025-11-23

**Authors:** Kian D. Samadian, Emma Chua, Boyu Peng, Adriana Coleska, Ahmad Hassan, Paul Chong, Brian Locke, David M. Liebovitz, Cory Rohlfsen, Shuhan He

**Affiliations:** 1 Department of Emergency Medicine, Massachusetts General Hospital, 55 Fruit St, Boston, MA 02114 USA; 2 Pasadena City College, Pasadena, CA USA; 3 ConductScience, Skokie, IL USA; 4 Department of Ophthalmology, Walter Reed Medical Center, Bethesda, MD USA; 5 Department of Pulmonary and Critical Care, Intermountain Medical Center, Murray, UT USA; 6 Feinberg School of Medicine, Northwestern University, Chicago, IL USA; 7 Department of General Internal Medicine, University of Nebraska Medical Center, Omaha, NE USA

**Keywords:** Shannon entropy, Diagnostic uncertainty, Clinical decision-making, Youden’s index, Information theory, Diagnostic performance, Health care, Mathematics and computing, Medical research

## Abstract

Interpreting clinical findings is fundamental to diagnosis and care. However, the contribution of individual features to reducing diagnostic uncertainty remains unclear. Information theory’s Shannon entropy offers a way to quantify how much a finding narrows diagnostic possibilities. We analyzed 405 symptoms, physical signs, demographic factors, and tests drawn from 23 reviews to calculate entropy reduction from diagnostic tables and compared them to established accuracy measures, including Youden’s index and predictive values. Most features yielded modest uncertainty reductions, with nearly half removing less than one-fifth of uncertainty, while a subset of high-performance findings reduced uncertainty by more than 40%. Entropy reduction correlated strongly with Youden’s index and positive predictive value. Entropy analysis may enhance evaluation by highlighting features that offer greater informational benefit.

**Supplementary Information:**

The online version contains supplementary material available at 10.1038/s41598-025-29069-0.

## Introduction

The use of clinical symptoms to guide medical decision-making is a core tenet of evidence-based practice and plays a critical role in reducing diagnostic error. In the U.S., diagnostic errors contribute to an estimated 98,000 deaths annually, as reported by the Institute of Medicine^1,2^. While much attention has been given to the accuracy of diagnostic tests, far less has focused on the interpretive value of symptoms themselves, which is the earliest and often most accessible data available to clinicians^3,4^. Traditional diagnostic metrics such as sensitivity, specificity, positive predictive value (PPV), and negative predictive value (NPV) provide essential insights into test performance, but do not fully capture how individual clinical findings contribute to narrowing a differential diagnosis^5–9^.

In real-world settings, particularly in emergency care, clinicians are often faced with complex, undifferentiated presentations. The ability to efficiently triage and prioritize diagnostic pathways depends heavily on early symptom interpretation. However, the diagnostic value of symptoms is variable, and some may contribute more to reducing uncertainty than others. Without a formal way to quantify how symptoms affect diagnostic reasoning, clinicians are left to rely on heuristics, experience, and often, extensive testing, a pattern that may contribute to over-testing, delayed diagnosis, and potential harm.

Shannon entropy, a foundational concept in information theory and machine learning, offers a novel solution to this problem^10^. Defined as a measure of uncertainty within a system, Shannon entropy can be applied to clinical scenarios in which the diagnostic uncertainty is highest upon presentation and gradually decreases as symptoms are assessed and interpreted^11–17^. While entropy has been widely used in theoretical modeling and decision tree analyses, its clinical application to quantify the informational value of symptoms remains underexplored^18–21^.

In a clinical context, higher entropy reflects greater diagnostic uncertainty, whereas a reduction in entropy indicates that a test result has helped clarify the patient’s condition. By applying Shannon entropy to standard 2 × 2 diagnostic tables, it becomes possible to derive an “entropy removal” metric, a direct measure of how much diagnostic uncertainty a given test result alleviates. This approach complements established measures like the area under the receiver operating characteristic curve (AUROC) or the number needed to diagnose (NND), and has the potential to offer a more intuitive framework for clinicians seeking to understand the real-world value of a diagnostic intervention.

In this study, we sought to (1) demonstrate how Shannon entropy can be computed from standard diagnostic metrics (true positives, false positives, true negatives, and false negatives), (2) evaluate how effectively these tests reduce diagnostic uncertainty by quantifying entropy removal, and (3) discuss the implications for clinical decision-making, including the limitations introduced by incomplete data and the potential for selection bias. By highlighting this new lens for test evaluation, we aim to inform future research on integrating entropy-based measures into systematic reviews, meta-analyses, and clinical guidelines. Ultimately, quantifying diagnostic “information gain” or “removed entropy” may represent the next logical step in advancing an evidence-based framework that better aligns with the nuanced realities of patient care.

## Results

A total of 405 distinct clinical features were evaluated across 23 eligible reviews encompassing likelihood ratios and a broad range of diseases (see Supplementary Table S1). These features included a variety of symptoms (e.g., chest pain characteristics, physical examination signs), a few key demographic factors (such as sex and age thresholds), and various diagnostic tests (e.g., radiographs, ultrasonography, point-of-care imaging). To quantify the information gain per feature, we applied our entropy-removal framework, derived from decision tree node-splitting theory. This calculation reflects the reduction in uncertainty that results from using a specific diagnostic feature to classify patients. Essentially, it analyzes how well each diagnostic sign or symptom is able to separate patients by those who have the condition and those who don’t.

Shannon entropy was computed for each diagnostic scenario, and the entropy removed by each feature (both the situational reduction and the percentage of removed entropy) was quantified alongside traditional 2 × 2 performance metrics (sensitivity, specificity, PPV, NPV, Youden’s index, and DOR). Supplementary Figure S1 displays the relationship between different metrics and entropy removal.

Overall, the majority of features presented only modest reductions in diagnostic uncertainty, with nearly half resulting in removed entropy values of less than 20%. Most values did match expectations: information gain ranged from near-zero for common risks or demographics (e.g., joint pain, male) to over 20–30% removed entropy for certain high-performance clinical findings. It is worth noting that while many single features have limited impact on their own, a subset of findings can substantially reduce uncertainty in diagnosis. Some of the largest reductions were seen not with symptoms alone, but alongside diagnostic tests, particularly imaging modalities such as bedside ultrasonography and radiographic evaluation.

Entropy removal showed statistically significant moderate positive correlations with all conventional accuracy metrics, with the strength of correlation varying with each. We observed that the two strongest positive correlations were between the percentage of entropy removed and Youden’s index (Pearson correlation coefficient *r* ≈ 0.89) as well as with the positive predictive value (PPV; *r* ≈ 0.74; *p* < 0.001 for both), depicted in Supplementary Figure S2. These were generally higher than correlations with the individual sensitivity or specificity alone.

Entropy removal showed the strongest association with Youden’s index and positive predictive value due to their shared dependence on sensitivity, specificity, and true-positive/true-negative performance. However, their difference lies in cases where prevalence or likelihood ratio differences alter the entropy calculation. Where accuracy metrics like sensitivity, specificity, or Youden’s index describe how well a test discriminates between disease and non-disease, entropy removal instead quantifies how much that discrimination actually reduces diagnostic uncertainty. For example, some acute myocardial cases ended with high J values, but their low prevalence resulted in merely modest entropy removal. Conversely, features with a large difference in sensitivity and specificity (one high and one low) could result in low J values but surprisingly high entropy removal, showing that accuracy alone may not fully capture informational contribution. Entropy reduction also exhibited a moderate correlation with sensitivity (*r* ≈ 0.70) and with the logged diagnostic odds ratio (DOR; *r* ≈ 0.63; *p* < 0.001 for both). By contrast, the correlation with specificity was weaker (*r* ≈ 0.50), and the weakest association was observed with negative predictive value (NPV; *r* ≈ 0.43). All of these correlations were highly significant (*p* < 0.001). These results indicate that the measure of uncertainty reduction captures performance most consistently with metrics that integrate both sensitivity and specificity (Youden Index: Sensitivity + Specificity − 1; PPV: TP/(TP + FP)).

**Fig. 1:**
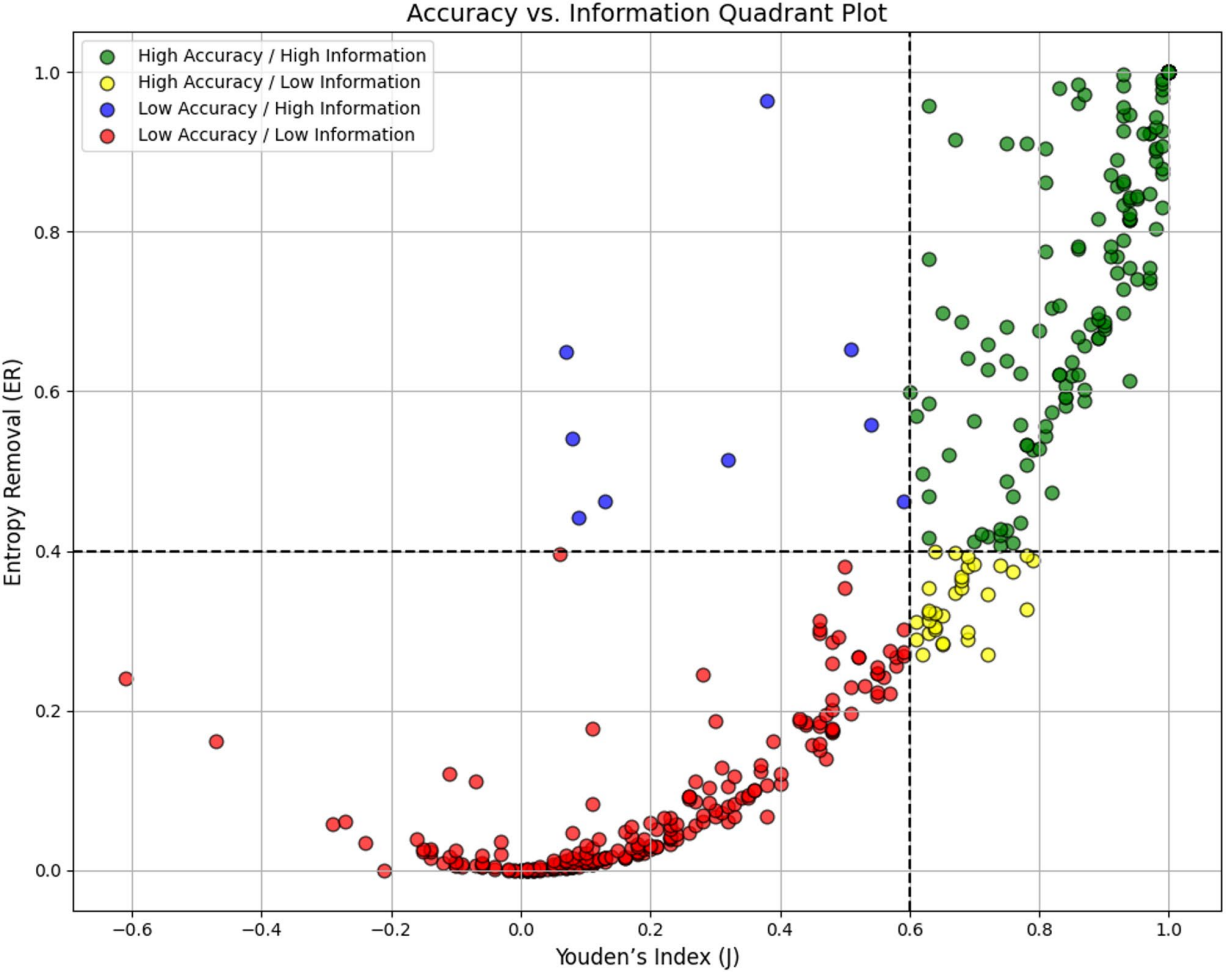
Accuracy vs. information quadrant plot. Depicting of removed entropy plotted by Youden’s index with cutlines at Youden’s J of 0.60 and entropy removal of 0.4 or 40% to divide data points into quadrants of diagnostic value.

Using threshold cut-offs of Youden’s J = 0.60 and entropy removal (ER) = 40%, the 405 clinical symptoms were further classified into four quadrants of diagnostic practicality: High Accuracy/High Information (J ≥ 0.60, ER ≥ 0.40), High Accuracy/Low Information (J ≥ 0.60, ER < 0.40), Low Accuracy/High Information (J < 0.60, ER ≥ 0.40), Low Accuracy/Low Information (J < 0.60, ER < 0.40). These categories were utilized to demonstrate a test or symptom’s information yield beyond simply their percentage of removed uncertainty, displayed by Fig. 1.

Out of 405 features, 145 fell into the High Accuracy/High Information category. Two of our highest-performing findings included the use of ocular ultrasounds, one in the diagnosis of retinal detachment, and the other in detecting raised intracranial pressure. Quite a few of our High Accuracy/High Information features involved ultrasonographic tests, with multiple even attaining J values of 1 and entropy removal values of 1, or 100% (see Table 1).

**Table 1:**
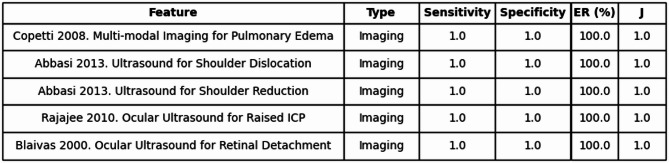
High Accuracy/High Information.

Shows the top five clinical features with both high diagnostic accuracy (Youden’s Index ≥ 0.60) and high entropy removal (ER ≥ 0.40, or 40%). Table includes feature, type, sensitivity, specificity, entropy removal percentage, and Youden’s Index (J).

*ICP* Intracranial Pressure.

The intermediate quadrants of High Accuracy/Low Information and Low Accuracy/High Information expose the imbalances between metrics. In such cases, the traditional accuracy metric of Youden’s Index contradicts the information level presented by the feature. For example, in High Accuracy/Low Information scenarios, a test may achieve excellent sensitivity and specificity (therein reflecting a high J) yet contribute surprisingly little to reducing uncertainty. 32 features fell into this category. An example is shown in Table 2, demonstrating a case covering acute myocardial infarction with a high Youden’s Index (J = 0.72) but a relatively low entropy removal value of 0.27, or 27%.

**Table 2:**
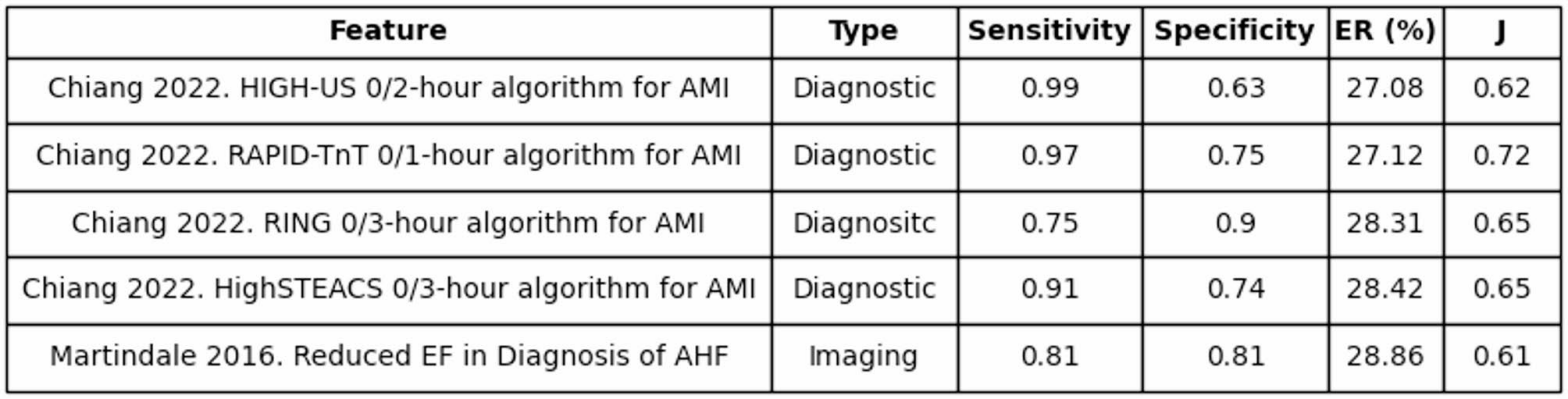
High Accuracy/Low Information.

Shows five clinical features with high diagnostic accuracy (Youden’s Index ≥ 0.60) and low entropy removal (ER < 0.40, or 40%). Table includes feature, type, sensitivity, specificity, entropy removal percentage, and Youden’s Index (J).

*AMI* Acute Myocardial Infarction, *EF* Ejection Fraction, *AHF* Acute Heart Failure.

Conversely, there were 9 scenarios that fell into the Low Accuracy/High Information category, entailing an unexceptional accuracy by Youden’s Index, while still being notably effective at reducing diagnostic uncertainty. Such incidents are often due to an extreme in one performance parameter (e.g., very high specificity resulting in a strong likelihood ratio despite mediocre sensitivity). Table 3 shows features that can exhibit a low J while still achieving a high entropy removal value in their respective diagnoses.

**Table 3:**
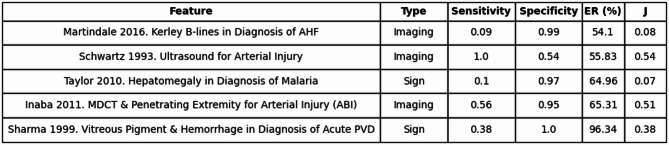
Low Accuracy/High Information.

Shows five clinical features with low diagnostic accuracy (Youden’s Index < 0.60) and high entropy removal (ER ≥ 0.40, or 40%). Table includes feature, type, sensitivity, specificity, entropy removal percentage, and Youden’s Index (J).

*AHF* Acute Heart Failure, *MDCT* Multidetector Computed Tomography, *ABI* Ankle-Brachial Index, *PVD* Posterior Vitreous Detachment.

For the remaining 219 features that fell within the final category of Low Accuracy/Low Information, their findings offered neither reliable accuracy nor meaningful information gain, contributing little to clinical decision-making. For example, a case on joint pain symptoms in the evaluation of malaria had a resultant J of −0.1 and an entropy removal value of 0.0001, or 0.01% (see Table 4). These metrics show that some symptoms may prove unhelpful in their respective diagnoses when evaluated alone.

**Table 4:**
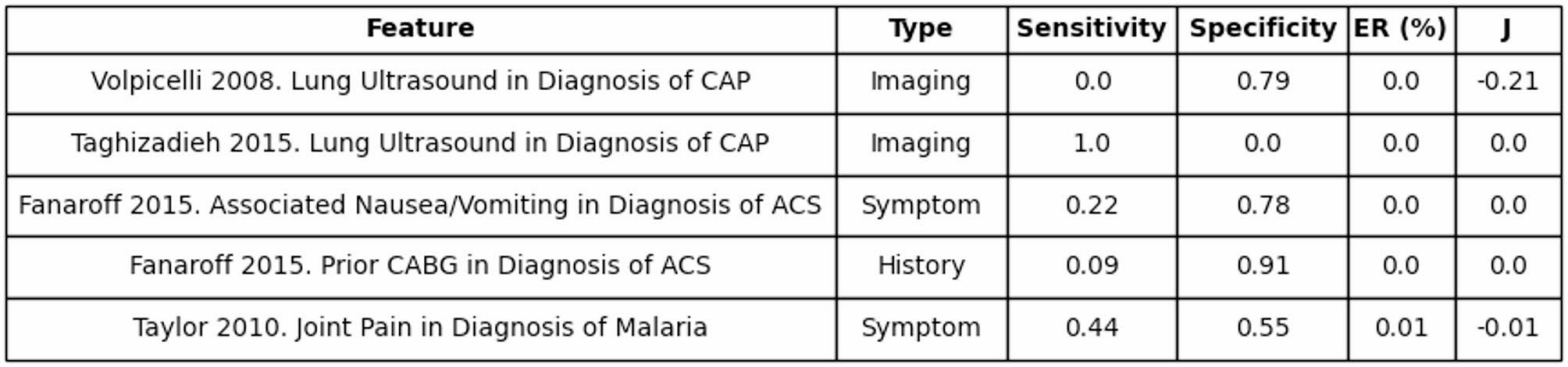
Low accuracy/low information.

Shows five clinical features with low diagnostic accuracy (Youden’s Index < 0.60) and low entropy removal (ER < 0.40, or 40%). Table includes feature, type, sensitivity, specificity, entropy removal percentage, and Youden’s Index (J).

*CAP* Community-acquired Pneumonia, *ACS* Acute Coronary Syndrome, *CABG* Coronary Artery Bypass Graft.

Of all 405 features analyzed, 3 were in the category of demographics, 160 in Imaging, 65 in Lab/Diagnostic Tests, and 177 in Signs/Symptoms/History (see Table 5).

**Table 5:**
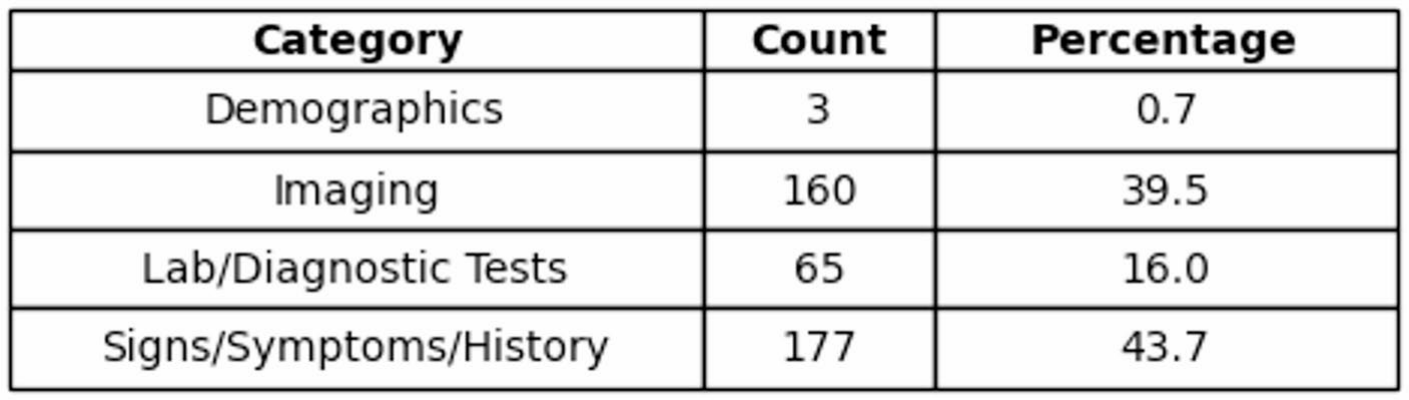
Overall category counts.

Displays the number of analyzed features grouped by category (demographics, signs/symptoms, laboratory tests, imaging). Table includes percentage distribution.

## Discussion

In this study, we applied Shannon entropy to quantify the informational value of clinical features (symptoms, physical exam findings, demographics, and diagnostic tests) across a broad array of diseases. By computing entropy removal from standard 2 × 2 diagnostic tables, we demonstrated how this metric complements traditional diagnostic performance measures and offers a novel perspective on the real-world value of clinical findings.

Overall, our findings indicate that most individual clinical features confer only modest reductions in diagnostic uncertainty, with nearly half removing less than 20% of baseline entropy. This observation aligns with clinical intuition: no single symptom is often definitive in isolation. However, a subset of features, particularly those with both high sensitivity and specificity, produced substantial entropy reductions, sometimes exceeding 60–70%. This reinforces the importance of high-performance findings in clinical reasoning and supports the continued reliance on aggregating multiple moderately informative features in practice. Notably, several of the most impactful features were not isolated symptoms but diagnostic tests, especially bedside ultrasonography. For example, ocular ultrasound for retinal detachment achieved near-complete entropy reduction (~ 100%), highlighting the power of targeted point-of-care imaging in time-sensitive scenarios^21^.

Entropy removal correlated most strongly with Youden’s index (*r* ≈ 0.89) and positive predictive value (*r* ≈ 0.74), both of which integrate aspects of true-positive and true-negative rates. Moderate correlations were also observed with sensitivity (*r* ≈ 0.70) and the logged diagnostic odds ratio (*r* ≈ 0.63), while weaker associations were found with specificity (*r* ≈ 0.50) and negative predictive value (*r* ≈ 0.43). These results suggest that entropy-based measures best reflect diagnostic tools that perform well across multiple axes, rather than excelling in a single domain.

Traditional accuracy metrics (sensitivity, specificity, Youden’s J) quantify a test’s discriminative performance but do not capture how much uncertainty they remove in practice. Entropy removal complements these metrics by measuring the proportional drop in diagnostic entropy a finding provides. Two tests may share the same J yet differ in entropy removal due to prevalence, asymmetry in false positives vs. false negatives, or context-specific likelihood ratios. Our quadrant framework highlights that high accuracy alone does not guarantee high information gain, and vice versa, thereby guiding clinicians to prioritize tests that both perform well and decisively narrow the differential.

We deliberately use the term “entropy removal” rather than “information gain” or “entropy reduction” to emphasize its clinical framing. In machine learning, “information gain” refers to a technical criterion for decision tree splitting, but that meaning does not translate well to bedside medicine, where tests may add data which can be overwhelming or tangential without reducing uncertainty. “Entropy removal” highlights how much a finding narrows the differential in ways that matter to clinicians: the value of a test is not in producing more information, but in reducing diagnostic uncertainty at the point of care.

To translate this clinically-oriented definition into practice, we developed a quadrant-based framework using Youden’s index and entropy removal as axes. Among 405 evaluated features, 145 fell into the “High Accuracy/High Information” quadrant: ideal findings that were both diagnostically accurate and significantly reduced uncertainty. These included ultrasonographic evaluations for retinal detachment and elevated intracranial pressure^22^. Conversely, the “Low Accuracy/Low Information” quadrant included many demographic traits and vague symptoms, such as vomiting or joint pain, which offered negligible entropy reductions^23,24^. The middle quadrants highlighted important mismatches. Some features with excellent sensitivity and specificity, like the Chiang 2022 RAPID-TnT algorithm, had relatively low entropy removal, perhaps due to modest shifts in disease probability or low pretest prevalence^25^. Others, like vitreous hemorrhage in posterior vitreous detachment, showed substantial entropy removal despite relatively low diagnostic accuracy, often driven by extremely high specificity^26^. These findings reveal that high accuracy does not always equate to high information gain, and vice versa.

Symptom-based findings generally outperformed demographics in entropy removal. For example, being male (as a risk factor for hemorrhagic stroke) reduced uncertainty by only 0.41%^27^. Similar patterns were observed for age thresholds and other nonspecific historical risk factors, which often removed less than 1% of entropy in their respective disease contexts. These results suggest that although demographics are statistically associated with disease, they rarely offer meaningful diagnostic clarity at the individual level.

By contrast, certain specific findings yielded substantial information gain. For example, a change in cardiac output in response to fluid administration produced a 68% reduction in entropy in assessing hypovolemia, with a diagnostic odds ratio of ~ 84^28^. Features with very high specificity generated large entropy reductions when present, while highly sensitive findings did so when absent, highlighting the complementary role of each in narrowing diagnostic possibilities. Unsurprisingly, many of the highest-performing findings came from ultrasonographic modalities used in procedural, anatomic, and physiologic contexts^29–38^. Across all conditions, features with strong likelihood ratios driven by high sensitivity, high specificity, or both tended to be the most informative^39–43^.

This study has several limitations. First, our analysis was restricted to the 23 reviews with sufficient data to compute entropy removal, raising the possibility of selection bias. Second, our model does not capture the joint or sequential impact of multiple features, which is more reflective of real-world diagnostic reasoning. Future research should apply entropy-based methods to multifactorial diagnostic models. Third, although entropy removal and Youden’s J share the same 2 × 2 contingency-table inputs, their high correlation partly reflects this shared foundation. Entropy removal, however, adds unique value by quantifying the reduction in diagnostic uncertainty, complementing rather than duplicating accuracy metrics. Lastly, all calculations rely on published performance metrics, which may not always reflect real-world test behavior due to population, setting, or operator variability. Despite these limitations, entropy removal offers a novel and intuitive method for evaluating the diagnostic utility of clinical findings. Future applications could include integrating entropy removal into clinical decision tools, likelihood ratio calculators, and diagnostic guidelines. Additionally, exploring the additive or interactive value of combinations of symptoms and tests may further improve diagnostic accuracy and resource utilization.

In conclusion, Shannon entropy may provide a clinically meaningful way to quantify how much a given feature reduces diagnostic uncertainty. By incorporating entropy removal alongside conventional test characteristics, clinicians can better identify which findings offer greater informational value, ultimately improving decision-making in high-stakes, resource-limited environments such as emergency care.

## Methods

Diagnostic metrics, meaning true positives (TP), false positives (FP), false negatives (FN), and true negatives (TN), were extracted from the NNT Database for Likelihood Ratio (LR) reviews, which contained 54 articles. Of the 54 reviews initially considered, 2 were identified as duplicates and removed. Among the remaining 52 studies, only 23 contained sufficient data to calculate the necessary values, as many lacked key information such as prevalence, sensitivity, or specificity. For most studies that did not explicitly report TP, FP, FN, or TN values, alternative values such as total study population (N), prevalence, sensitivity, and specificity were recorded. Then, those values were used to derive TP, FP, FN, and TN using the standard formulas listed below.

True Positives (TP): Prevalence Sensitivity Total Patients.

False Positives (FP): (1- Prevalence) (1- Specificity) Total Patients.

False Negatives (FN): Prevalence (1- Specificity) Total Patients.

True Negatives (TN): (1- Prevalence) Specificity Total Patients.

Data collection and computation were performed from February 5th, 2025, to March 2nd, 2025. All data was compiled in Google Sheets, where formulas were applied to calculate missing values. The finalized dataset was processed in a separate sheet using additional formulas to compute Shannon entropy and entropy removal. Using the diagnostic metrics (TP, FP, FN, etc.), Shannon entropy was calculated using the equations below. The parent nodes refer to the total sample N, and the child nodes refer to the positive (TP, FP) and negative (FN, TN) tests. The representations for the number of positive and negative tests are n_positive_ and n_negative_, respectively.


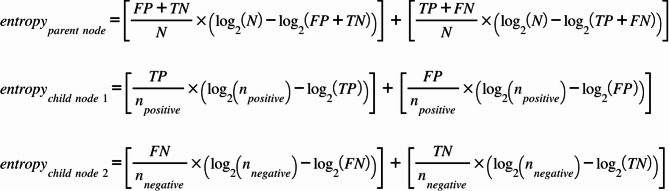


Entropy removal was then calculated using the following equation. Entropy removal corresponds to the difference between the entropy of the parent node (the total entropy of the system) and the weighted average entropy of the children nodes (proportional to n_positive_ and n_negative_, respectively).


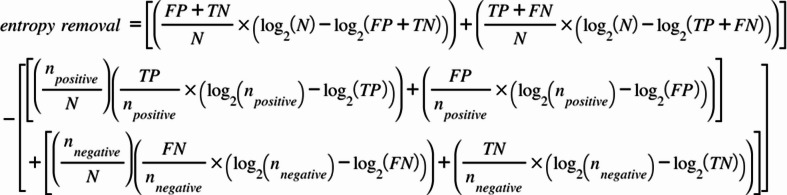


The clinical features were categorized into one of four diagnostic utility quadrants using prespecified threshold cutoffs for both Youden’s Index (J) and entropy removal (ER) value. For this analysis, we selected J ≥ 0.60 to define high accuracy and ER ≥ 0.40 to define high information gain. Based on these cutoffs, each finding was sorted into its respective quadrant and analyzed to identify discordance between traditional accuracy and information gain.

All statistical analyses were performed in Google Sheets. The number of individual clinical features evaluated totaled 405 (*n* = 405). For each feature, entropy reduction was calculated from published diagnostic tables and compared to the following accuracy measures: sensitivity, specificity, positive predictive value (PPV), negative predictive value (NPV), Youden’s Index (J), and diagnostic odds ratio (DOR). Associations between entropy reduction and each measure were quantified via the two-tailed Pearson correlation coefficient. Each p-value was found to be smaller than the precision limit of Google Sheet’s statistical functions, and as such, exact results are reported as *p* < 0.0001. Statistical significance was defined as *p* < 0.05. No adjustments for multiple comparisons were applied, as analyses were exploratory and descriptive in nature. Additional figures are available in the Supplementary Information.

For purpose of reproducibility, the code used for figure creation, detailed instructions on the steps of computation, and all source data/results through analysis are publicly available at https://github.com/emma-r-chua/Entropy-Paper-Code-and-Source-Data.git.

## Supplementary Information

Below is the link to the electronic supplementary material.

Supplementary Material 1

## Data Availability

The datasets generated and analyzed in the study are available upon request. All data were obtained from the NNT Likelihood Ratio Database (https:/thennt.com). Source data underlying the figures and tables are provided with this paper.
