# Supplementary material for: Entropy removal of clinical features
Source: Sci Rep. 2025 Nov 23;15:45182. doi: 10.1038/s41598-025-29069-0 (PMC12749281; doi:10.1038/s41598-025-29069-0)
Supplement: Supplementary file 1 — Supplementary Material 1 [file 41598_2025_29069_MOESM1_ESM.docx]

Supplementary Materials for
**Entropy Removal of Clinical Features**

Kian D. Samadian MD, Emma Chua BS, Boyu Peng MS, Adriana Coleska MD MBA, Ahmad Hassan MD, Paul Chong MD, Brian Locke MD, Shuhan He MD

Corresponding author: Kian D. Samadian MD ([ksamadian@mgb.org](mailto:ksamadian@mgb.org))

**This file includes:**

Supplementary Table S1 and Supplementary Figures S1 and S2.

| **Feature** | **Category** | **Sensitivity** | **Specificity** | **Youden's Index (J)** | **Entropy Removal (%)** |
| --- | --- | --- | --- | --- | --- |
| Taghizadieh 2015. Lung Ultrasound in Diagnosis of CAP | Imaging | 1.00 | 0.00 | 0.00 | 0.00% |
| Volpicelli 2008. Lung Ultrasound in Diagnosis of CAP | Imaging | 0.00 | 0.79 | -0.21 | 0.00% |
| Fanaroff 2015. Associated Nausea/Vomiting in Diagnosis of ACS | Signs/Symptoms/History | 0.22 | 0.78 | 0.00 | 0.00% |
| Fanaroff 2015. Prior CABG in Diagnosis of ACS | Signs/Symptoms/History | 0.09 | 0.91 | 0.00 | 0.00% |
| Fanaroff 2015. Family history of CAD in Diagnosis of ACS | Signs/Symptoms/History | 0.37 | 0.64 | 0.01 | 0.01% |
| Taylor 2010. Joint Pain in Diagnosis of Malaria | Signs/Symptoms/History | 0.44 | 0.55 | -0.01 | 0.01% |
| Runchey 2010. Plantar response: single extensor in Diagnosis of Hemorrhagic Stroke | Signs/Symptoms/History | 0.62 | 0.39 | 0.01 | 0.01% |
| Jaffe 1968. Floaters and Flashes in Diagnosis of Acute PVD | Signs/Symptoms/History | 0.42 | 0.60 | 0.01 | 0.01% |
| Taylor 2010. Tongue pallor (Adults Only) in Diagnosis of Malaria | Signs/Symptoms/History | 0.08 | 0.91 | -0.01 | 0.01% |
| Byer 1994. Floaters and Flashes in Diagnosis of Acute PVD | Signs/Symptoms/History | 0.55 | 0.47 | 0.02 | 0.02% |
| Taylor 2010. Dyspnea (Children Only) in Diagnosis of Malaria | Signs/Symptoms/History | 0.34 | 0.64 | -0.02 | 0.03% |
| Brod 1991. Floaters and Flashes in Diagnosis of Acute PVD | Signs/Symptoms/History | 0.64 | 0.38 | 0.02 | 0.03% |
| Fanaroff 2015. Burning pain in Diagnosis of ACS | Signs/Symptoms/History | 0.14 | 0.88 | 0.02 | 0.05% |
| Fanaroff 2015. Any tobacco use in Diagnosis of ACS | Signs/Symptoms/History | 0.38 | 0.65 | 0.03 | 0.06% |
| Fanaroff 2015. Radiation to right arm in Diagnosis of ACS | Signs/Symptoms/History | 0.05 | 0.96 | 0.01 | 0.06% |
| Taylor 2010. Palmar pallor (Adults Only) in Diagnosis of Malaria | Signs/Symptoms/History | 0.09 | 0.89 | -0.02 | 0.07% |
| Taylor 2010. Joint pain (Children Only) in Diagnosis of Malaria | Signs/Symptoms/History | 0.12 | 0.91 | 0.03 | 0.11% |
| Taylor 2010. Hepatomegaly (Adults Only) in Diagnosis of Malaria | Signs/Symptoms/History | 0.01 | 1.00 | 0.01 | 0.11% |
| Runchey 2010. Hemiparesis in Diagnosis of Hemorrhagic Stroke | Signs/Symptoms/History | 0.63 | 0.33 | -0.04 | 0.11% |
| Martindale 2016. Dyspnea at rest in Diagnosis of AHF | Signs/Symptoms/History | 0.55 | 0.50 | 0.04 | 0.13% |
| Boldrey 1983. Floaters and Flashes in Diagnosis of Acute PVD | Signs/Symptoms/History | 0.52 | 0.53 | 0.05 | 0.14% |
| Fanaroff 2015. Tachycardia (heart rate>120) in Diagnosis of ACS | Signs/Symptoms/History | 0.04 | 0.98 | 0.02 | 0.16% |
| Fanaroff 2015. Cerebrovascular disease in Diagnosis of ACS | Signs/Symptoms/History | 0.10 | 0.93 | 0.03 | 0.18% |
| Martindale 2016. ST-depression in Diagnosis of AHF | Lab/Diagnostic Tests | 0.06 | 0.97 | 0.02 | 0.18% |
| Taylor 2010. Splenomegaly (Adults Only) in Diagnosis of Malaria | Signs/Symptoms/History | 0.01 | 1.00 | 0.01 | 0.19% |
| Fanaroff 2015. Any improvement with nitroglycerin in Diagnosis of ACS | Signs/Symptoms/History | 0.71 | 0.35 | 0.06 | 0.24% |
| Taylor 2010. Vomiting (Adults Only) in Diagnosis of Malaria | Signs/Symptoms/History | 0.15 | 0.90 | 0.05 | 0.36% |
| Fanaroff 2015. Associated diaphoresis in Diagnosis of ACS | Signs/Symptoms/History | 0.26 | 0.81 | 0.07 | 0.36% |
| Taylor 2010. Headache (Adults Only) in Diagnosis of Malaria | Signs/Symptoms/History | 0.80 | 0.27 | 0.07 | 0.37% |
| Taylor 2010. Headache (All Participants) in Diagnosis of Malaria | Signs/Symptoms/History | 0.78 | 0.29 | 0.07 | 0.40% |
| Fanaroff 2015. Obesity in Diagnosis of ACS | Signs/Symptoms/History | 0.40 | 0.68 | 0.08 | 0.41% |
| Runchey 2010. Male in Diagnosis of Hemorrhagic Stroke | Demographics | 0.57 | 0.51 | 0.08 | 0.41% |
| Runchey 2010. Hypertension in Diagnosis of Hemorrhagic Stroke | Signs/Symptoms/History | 0.68 | 0.40 | 0.08 | 0.44% |
| Taylor 2010. Diarrhea (Children Only) in Diagnosis of Malaria | Signs/Symptoms/History | 0.13 | 0.81 | -0.06 | 0.44% |
| Taylor 2010. Diarrhea (Adults Only) in Diagnosis of Malaria | Signs/Symptoms/History | 0.03 | 0.93 | -0.04 | 0.44% |
| Fanaroff 2015. Abrupt onset in Diagnosis of ACS | Signs/Symptoms/History | 0.76 | 0.32 | 0.08 | 0.46% |
| Taylor 2010. Diarrhea (All Participants) in Diagnosis of Malaria | Signs/Symptoms/History | 0.11 | 0.83 | -0.06 | 0.47% |
| Fanaroff 2015. Lung rales in Diagnosis of ACS | Signs/Symptoms/History | 0.10 | 0.95 | 0.05 | 0.49% |
| Fanaroff 2015. Hypotension (SBP<100) in Diagnosis of ACS | Signs/Symptoms/History | 0.04 | 0.99 | 0.03 | 0.50% |
| Fanaroff 2015. Hyperlipidemia in Diagnosis of ACS | Signs/Symptoms/History | 0.42 | 0.67 | 0.09 | 0.51% |
| Fanaroff 2015. Tachypnea in Diagnosis of ACS | Signs/Symptoms/History | 0.10 | 0.95 | 0.05 | 0.51% |
| Taylor 2010. Cough (All Participants) in Diagnosis of Malaria | Signs/Symptoms/History | 0.41 | 0.50 | -0.09 | 0.52% |
| Fanaroff 2015. Diabetes in Diagnosis of ACS | Signs/Symptoms/History | 0.26 | 0.82 | 0.08 | 0.57% |
| Fanaroff 2015. Radiation to neck or jaw in Diagnosis of ACS | Signs/Symptoms/History | 0.24 | 0.84 | 0.08 | 0.61% |
| Runchey 2010. Cigarette smoking in Diagnosis of Hemorrhagic Stroke | Signs/Symptoms/History | 0.38 | 0.52 | -0.10 | 0.63% |
| Martindale 2016. Absence of productive cough in Diagnosis of AHF | Signs/Symptoms/History | 0.82 | 0.26 | 0.08 | 0.63% |
| Tanner 2000. Floaters and Flashes in Diagnosis of Acute PVD | Signs/Symptoms/History | 0.36 | 0.54 | -0.10 | 0.64% |
| Fanaroff 2015. Associated syncope in Diagnosis of ACS | Signs/Symptoms/History | 0.09 | 0.84 | -0.07 | 0.64% |
| Fanaroff 2015. Peripheral arterial disease in Diagnosis of ACS | Signs/Symptoms/History | 0.08 | 0.97 | 0.05 | 0.70% |
| Taylor 2010. Chills/rigors (Adults Only) in Diagnosis of Malaria | Signs/Symptoms/History | 0.78 | 0.32 | 0.10 | 0.70% |
| Fanaroff 2015. Hypertension in Diagnosis of ACS | Signs/Symptoms/History | 0.59 | 0.52 | 0.11 | 0.71% |
| Martindale 2016. Hyperlipidemia in Diagnosis of AHF | Signs/Symptoms/History | 0.34 | 0.75 | 0.09 | 0.72% |
| Runchey 2010. Diabetes mellitus in Diagnosis of Hemorrhagic Stroke | Signs/Symptoms/History | 0.17 | 0.74 | -0.09 | 0.74% |
| Runchey 2010. Atrial fibrillation in Diagnosis of Hemorrhagic Stroke | Signs/Symptoms/History | 0.04 | 0.90 | -0.06 | 0.76% |
| Hikichi and Trempe 1994. Floaters and Flashes in Diagnosis of Acute PVD | Signs/Symptoms/History | 0.54 | 0.57 | 0.11 | 0.78% |
| Fanaroff 2015. Prior myocardial infarction in Diagnosis of ACS | Signs/Symptoms/History | 0.28 | 0.82 | 0.10 | 0.87% |
| Martindale 2016. T-wave inversion in Diagnosis of AHF | Lab/Diagnostic Tests | 0.10 | 0.96 | 0.06 | 0.87% |
| Runchey 2010. Peripheral artery disease in Diagnosis of Hemorrhagic Stroke | Signs/Symptoms/History | 0.03 | 0.91 | -0.06 | 0.94% |
| Taylor 2010. Cough (Children Only) in Diagnosis of Malaria | Signs/Symptoms/History | 0.48 | 0.40 | -0.12 | 0.95% |
| Tabotabo 1980. Floaters and Flashes in Diagnosis of Acute PVD | Signs/Symptoms/History | 0.43 | 0.70 | 0.13 | 1.05% |
| Martindale 2016. Hepatojugular reflex in Diagnosis of AHF | Signs/Symptoms/History | 0.14 | 0.93 | 0.07 | 1.05% |
| Runchey 2010. Plantar response: both extensor in Diagnosis of Hemorrhagic Stroke | Signs/Symptoms/History | 0.16 | 0.92 | 0.08 | 1.08% |
| Strebel 2001. Inspiratory whoop in Diagnosis of Pertussis | Signs/Symptoms/History | 0.26 | 0.85 | 0.11 | 1.11% |
| Fanaroff 2015. “Typical” radiation in Diagnosis of ACS | Signs/Symptoms/History | 0.29 | 0.83 | 0.11 | 1.11% |
| Martindale 2016. DM in Diagnosis of AHF | Signs/Symptoms/History | 0.29 | 0.82 | 0.11 | 1.11% |
| Runchey 2010. Prior stroke in Diagnosis of Hemorrhagic Stroke | Signs/Symptoms/History | 0.11 | 0.79 | -0.10 | 1.13% |
| Harnden 2006. Paroxysmal cough in Diagnosis of Pertussis | Signs/Symptoms/History | 0.86 | 0.24 | 0.10 | 1.14% |
| Taylor 2010. Vomiting (All Participants) in Diagnosis of Malaria | Signs/Symptoms/History | 0.33 | 0.79 | 0.12 | 1.22% |
| Fanaroff 2015. Radiation to both arms in Diagnosis of ACS | Signs/Symptoms/History | 0.11 | 0.96 | 0.07 | 1.22% |
| Martindale 2016. Murmur in Diagnosis of AHF | Signs/Symptoms/History | 0.28 | 0.83 | 0.11 | 1.27% |
| Martindale 2016. Alveolar edema in Diagnosis of AHF | Signs/Symptoms/History | 0.06 | 0.99 | 0.05 | 1.31% |
| Martindale 2016. Pleural effusion in Diagnosis of AHF (Radiograph/Electrocardiogram Findings) | Imaging | 0.16 | 0.93 | 0.09 | 1.44% |
| Fanaroff 2015. Pleuritic pain in Diagnosis of ACS | Signs/Symptoms/History | 0.27 | 0.85 | 0.12 | 1.48% |
| Martindale 2016. Atrial fibrillation in Diagnosis of AHF | Signs/Symptoms/History | 0.20 | 0.90 | 0.10 | 1.50% |
| Martindale 2016. No history of COPD in Diagnosis of AHF | Signs/Symptoms/History | 0.79 | 0.34 | 0.13 | 1.52% |
| Fanaroff 2015. Men (as a Risk Factor) in Diagnosis of ACS | Demographics | 0.66 | 0.50 | 0.16 | 1.53% |
| Fanaroff 2015. Abnormal prior stress in Diagnosis of ACS | Signs/Symptoms/History | 0.12 | 0.96 | 0.08 | 1.55% |
| Taylor 2010. Vomiting (Children Only) in Diagnosis of Malaria | Signs/Symptoms/History | 0.32 | 0.81 | 0.13 | 1.56% |
| Fanaroff 2015. Change in pattern over prior 24 hours in Diagnosis of ACS | Signs/Symptoms/History | 0.27 | 0.86 | 0.13 | 1.62% |
| Park 2005. Posttussive emesis in Diagnosis of Pertussis | Signs/Symptoms/History | 0.33 | 0.83 | 0.16 | 1.63% |
| Martindale 2016. Wheezing in Diagnosis of AHF | Signs/Symptoms/History | 0.22 | 0.64 | -0.14 | 1.64% |
| Runchey 2010. Coronary artery disease in Diagnosis of Hemorrhagic Stroke | Signs/Symptoms/History | 0.06 | 0.83 | -0.11 | 1.68% |
| Taylor 2010. Chills/rigors (All Participants) in Diagnosis of Malaria | Signs/Symptoms/History | 0.54 | 0.62 | 0.16 | 1.71% |
| Runchey 2010. Level of consciousness:drowsy in Diagnosis of Hemorrhagic Stroke | Signs/Symptoms/History | 0.32 | 0.82 | 0.14 | 1.78% |
| Runchey 2010. Cervical bruit in Diagnosis of Hemorrhagic Stroke | Signs/Symptoms/History | 0.01 | 0.93 | -0.06 | 1.80% |
| Runchey 2010. Seizures accompanying neurologic deficit in Diagnosis of Hemorrhagic Stroke | Signs/Symptoms/History | 0.09 | 0.98 | 0.07 | 1.88% |
| Taylor 2010. Headache (Children Only) in Diagnosis of Malaria | Signs/Symptoms/History | 0.63 | 0.55 | 0.18 | 2.01% |
| Fanaroff 2015. Associated palpitations in Diagnosis of ACS | Signs/Symptoms/History | 0.06 | 0.91 | -0.03 | 2.10% |
| Diamond 1992. Floaters and Flashes in Diagnosis of Acute PVD | Signs/Symptoms/History | 0.55 | 0.64 | 0.19 | 2.14% |
| Runchey 2010. Kernig sign, Brudzinski sign, or both in Diagnosis of Hemorrhagic Stroke | Signs/Symptoms/History | 0.13 | 0.96 | 0.09 | 2.19% |
| Runchey 2010. Alcohol consumption in Diagnosis of Hemorrhagic Stroke | Signs/Symptoms/History | 0.48 | 0.70 | 0.18 | 2.21% |
| Martindale 2016. Renal Failure in Diagnosis of AHF | Signs/Symptoms/History | 0.15 | 0.95 | 0.10 | 2.22% |
| Martindale 2016. HTN in Diagnosis of AHF | Signs/Symptoms/History | 0.67 | 0.51 | 0.18 | 2.30% |
| Runchey 2010. Prior transient ischemic attack in Diagnosis of Hemorrhagic Stroke | Signs/Symptoms/History | 0.07 | 0.79 | -0.14 | 2.30% |
| Runchey 2010. Plantar response: both flexor in Diagnosis of Hemorrhagic Stroke | Signs/Symptoms/History | 0.11 | 0.74 | -0.15 | 2.41% |
| Martindale 2016. AFIB in Diagnosis of AHF | Signs/Symptoms/History | 0.30 | 0.85 | 0.15 | 2.51% |
| Hadeel and Dien 2013. Chest Radiographs in the Diagnosis of Pneumonia | Imaging | 0.90 | 0.00 | -0.10 | 2.58% |
| Taylor 2010. Chills/rigors (Children Only) in Diagnosis of Malaria | Signs/Symptoms/History | 0.49 | 0.70 | 0.19 | 2.59% |
| Fanaroff 2015. Pain reproduced on palpation in Diagnosis of ACS | Signs/Symptoms/History | 0.06 | 0.80 | -0.14 | 2.61% |
| Runchey 2010. Age <= 60 in Diagnosis of Hemorrhagic Stroke | Demographics | 0.50 | 0.70 | 0.20 | 2.62% |
| Runchey 2010. Hyperlipidemia in Diagnosis of Hemorrhagic Stroke | Signs/Symptoms/History | 0.07 | 0.78 | -0.15 | 2.64% |
| Taylor 2010. Hepatomegaly (Children Only) in Diagnosis of Malaria | Signs/Symptoms/History | 0.15 | 0.96 | 0.11 | 2.77% |
| Taylor 2010. Pallor (Children Only) in Diagnosis of Malaria | Signs/Symptoms/History | 0.33 | 0.84 | 0.17 | 2.82% |
| Luri 2009. Chest Radiographs in the Diagnosis of CAP | Imaging | 0.71 | 0.50 | 0.21 | 2.91% |
| Fanaroff 2015. Prior CAD in Diagnosis of ACS | Signs/Symptoms/History | 0.41 | 0.79 | 0.20 | 2.92% |
| Brod 1991. Vitreous Hemorrhage In Diagnosis of Acute PVD | Signs/Symptoms/History | 0.50 | 0.71 | 0.21 | 2.95% |
| Taylor 2010. Nausea (Children Only) in Diagnosis of Malaria | Signs/Symptoms/History | 0.36 | 0.82 | 0.18 | 3.02% |
| Martindale 2016. S3 in Diagnosis of AHF | Signs/Symptoms/History | 0.13 | 0.98 | 0.10 | 3.15% |
| Martindale 2016. PND in Diagnosis of AHF | Signs/Symptoms/History | 0.46 | 0.74 | 0.20 | 3.21% |
| Martindale 2016. Ischemic changes in Diagnosis of AHF | Signs/Symptoms/History | 0.34 | 0.84 | 0.18 | 3.29% |
| Strebel 2001. Posttussive emesis in Diagnosis of Pertussis | Signs/Symptoms/History | 0.56 | 0.68 | 0.23 | 3.37% |
| Runchey 2010. Acute onset of deficit in Diagnosis of Hemorrhagic Stroke | Signs/Symptoms/History | 0.44 | 0.32 | -0.24 | 3.49% |
| Martindale 2016. ST-elevation in Diagnosis of AHF | Lab/Diagnostic Tests | 0.05 | 0.92 | -0.03 | 3.58% |
| Martindale 2016. History of MI in Diagnosis of AHF | Signs/Symptoms/History | 0.32 | 0.87 | 0.19 | 3.85% |
| Martindale 2016. Orthopnea in Diagnosis of AHF | Signs/Symptoms/History | 0.52 | 0.70 | 0.23 | 3.86% |
| Man 2017. Lung Ultrasound in Diagnosis of Pneumonia in Childhood | Imaging | 0.79 | 0.44 | 0.24 | 3.88% |
| Runchey 2010. Atrial fibrillation on electrocardiogram in Diagnosis of Hemorrhagic Stroke | Lab/Diagnostic Tests | 0.02 | 0.82 | -0.16 | 3.91% |
| Strebel 2001. Paroxysmal cough in Diagnosis of Pertussis | Signs/Symptoms/History | 1.00 | 0.12 | 0.12 | 3.99% |
| Harnden 2006. Inspiratory whoop in Diagnosis of Pertussis | Signs/Symptoms/History | 0.50 | 0.73 | 0.23 | 4.10% |
| Martindale 2016. CAD in Diagnosis of AHF | Signs/Symptoms/History | 0.47 | 0.76 | 0.23 | 4.17% |
| Taylor 2010. Splenomegaly (All Participants) in Diagnosis of Malaria | Signs/Symptoms/History | 0.24 | 0.93 | 0.17 | 4.22% |
| Smith-Bindman 2014. Ultrasound for Diagnosis of Nephrolithiasis | Imaging | 0.49 | 0.75 | 0.24 | 4.50% |
| Yilmaz 2017. Lung Ultrasound in Diagnosis of Pneumonia in Childhood | Imaging | 0.96 | 0.12 | 0.08 | 4.71% |
| Fanaroff 2015. Pain similar to prior ischemia in Diagnosis of ACS | Signs/Symptoms/History | 0.47 | 0.79 | 0.26 | 4.76% |
| Byer 1994. Vitreous Hemorrhage In Diagnosis of Acute PVD | Signs/Symptoms/History | 0.20 | 0.96 | 0.16 | 4.90% |
| Martindale 2016. Arrhythmia in Diagnosis of AHF | Signs/Symptoms/History | 0.38 | 0.85 | 0.23 | 5.12% |
| Taylor 2010. Splenomegaly (Children Only) in Diagnosis of Malaria | Signs/Symptoms/History | 0.31 | 0.90 | 0.21 | 5.12% |
| Runchey 2010. Neck stiffness in Diagnosis of Hemorrhagic Stroke | Signs/Symptoms/History | 0.20 | 0.97 | 0.17 | 5.57% |
| Fanaroff 2015. Ischemic ECG in Diagnosis of ACS | Lab/Diagnostic Tests | 0.32 | 0.91 | 0.23 | 5.70% |
| Martindale 2016. Leg edema in Diagnosis of AHF | Signs/Symptoms/History | 0.52 | 0.75 | 0.27 | 5.71% |
| Taylor 2010. Dyspnea (Adults Only) in Diagnosis of Malaria | Signs/Symptoms/History | 0.17 | 0.54 | -0.29 | 5.77% |
| Martindale 2016. JVD in Diagnosis of AHF | Signs/Symptoms/History | 0.37 | 0.87 | 0.24 | 5.83% |
| Fanaroff 2015. ST depression in Diagnosis of ACS | Lab/Diagnostic Tests | 0.25 | 0.95 | 0.20 | 5.98% |
| Fanaroff 2015. “Typical” chest pain in Diagnosis of ACS | Signs/Symptoms/History | 0.66 | 0.66 | 0.32 | 6.15% |
| Runchey 2010. Headache in Diagnosis of Hemorrhagic Stroke | Signs/Symptoms/History | 0.46 | 0.82 | 0.28 | 6.16% |
| Martindale 2016. Normal sinus rhythm in Diagnosis of AHF | Signs/Symptoms/History | 0.55 | 0.18 | -0.27 | 6.19% |
| Martindale 2016. CRI in Diagnosis of AHF | Signs/Symptoms/History | 0.32 | 0.91 | 0.23 | 6.53% |
| Daniels 2016. Accuracy of POCUS with Moderate or Greater Hydronephrosis in Diagnosis of Nephrolithiasis | Imaging | 0.28 | 0.94 | 0.22 | 6.59% |
| Richardson 1999. Floaters and Flashes in The Diagnosis of Acute PVD | Signs/Symptoms/History | 0.64 | 0.69 | 0.33 | 6.75% |
| Park 2005. Inspiratory whoop in Diagnosis of Pertussis | Signs/Symptoms/History | 0.67 | 0.72 | 0.38 | 6.77% |
| Martindale 2016. Rales in Diagnosis of AHF | Signs/Symptoms/History | 0.62 | 0.68 | 0.30 | 6.79% |
| Runchey 2010. Loss of consciousness in Diagnosis of Hemorrhagic Stroke | Signs/Symptoms/History | 0.47 | 0.82 | 0.28 | 6.89% |
| Harnden 2006. Posttussive emesis in Diagnosis of Pertussis | Signs/Symptoms/History | 0.70 | 0.61 | 0.31 | 7.15% |
| Woo 2016. Point of Care Ocular Ultrasound for Diagnosis of Retinal Detatchment | Imaging | 0.48 | 0.82 | 0.30 | 7.52% |
| Schwartz 1993. Ultrasound and Penetrating Extremity Trauma in Diagnosis of Arterial Injury (Performance of Hard Signs) | Imaging | 0.49 | 0.83 | 0.32 | 8.00% |
| Ambroggio 2016. Lung Ultrasound in Diagnosis of Pneumonia in Childhood | Imaging | 0.58 | 0.75 | 0.33 | 8.29% |
| Fanaroff 2015. T wave inversion in Diagnosis of ACS | Lab/Diagnostic Tests | 0.24 | 0.87 | 0.11 | 8.36% |
| Martindale 2016. Elecsys Roche diagnostic 300 in Diagnosis of AHF | Lab/Diagnostic Tests | 0.90 | 0.38 | 0.29 | 8.42% |
| Runchey 2010. Vomiting in Diagnosis of Hemorrhagic Stroke | Signs/Symptoms/History | 0.34 | 0.93 | 0.27 | 8.71% |
| Herbst 2014. Accuracy of POCUS with Moderate or Greater Hydronephrosis in Diagnosis of Nephrolithiasis | Imaging | 0.31 | 0.95 | 0.26 | 8.94% |
| Chiang 2022. UTROPIA (55) 0/3-hour algorithm for Rapid Triage of AMI | Lab/Diagnostic Tests | 0.94 | 0.39 | 0.34 | 9.10% |
| Martindale 2016. Pleural effusion in Diagnosis of AHF (Ultrasound findings) | Imaging | 0.63 | 0.72 | 0.35 | 9.17% |
| Martindale 2016. Interstitial edema in Diagnosis of AHF | Signs/Symptoms/History | 0.31 | 0.95 | 0.26 | 9.23% |
| Chiang 2022. Nuremberg (40) 0/3-hour algorithm for Rapid Triage of AMI | Lab/Diagnostic Tests | 1.00 | 0.26 | 0.26 | 9.33% |
| Park 2005. Paroxysmal cough in Diagnosis of Pertussis | Signs/Symptoms/History | 1.00 | 0.35 | 0.35 | 9.49% |
| Martindale 2016. Enlarged cardiac silhouette in Diagnosis of AHF | Imaging | 0.75 | 0.62 | 0.36 | 9.98% |
| Martindale 2016. CHF in Diagnosis of AHF | Signs/Symptoms/History | 0.55 | 0.80 | 0.36 | 10.13% |
| Runchey 2010. Level of consciousness:coma in Diagnosis of Hemorrhagic Stroke | Signs/Symptoms/History | 0.35 | 0.94 | 0.29 | 10.37% |
| Zhan 2016. Lung Ultrasound in Diagnosis of Pneumonia in Childhood | Imaging | 0.40 | 0.91 | 0.32 | 10.49% |
| Bentzer 2016. Hemodynamically unstable, Central venous pressure in Response to Bolus of Intravenous Fluids | Signs/Symptoms/History | 0.62 | 0.76 | 0.38 | 10.72% |
| Kanski 1975. Vitreous Hemorrhage In Diagnosis of Acute PVD | Signs/Symptoms/History | 0.62 | 0.78 | 0.40 | 10.77% |
| Taylor 2010. Cough (Adults Only) in Diagnosis of Malaria | Signs/Symptoms/History | 0.35 | 0.58 | -0.07 | 11.12% |
| Marin 2013. Point of Care Ultrasonography for the Diagnosis of Abscess | Imaging | 0.97 | 0.30 | 0.27 | 11.16% |
| Moore 2007. Fluid in pelvis for any ectopic preg in Predicting Need for Operative Intervention | Signs/Symptoms/History | 0.39 | 0.94 | 0.33 | 11.82% |
| Taylor 2010. Dyspnea (All Participants) in Diagnosis of Malaria | Signs/Symptoms/History | 0.28 | 0.61 | -0.11 | 12.05% |
| Daniels 2016. Ultrasound for Diagnosis of Nephrolithiasis | Imaging | 0.65 | 0.75 | 0.40 | 12.11% |
| Runchey 2010. Diastolic blood pressure >110 mm Hg in Diagnosis of Hemorrhagic Stroke | Signs/Symptoms/History | 0.48 | 0.89 | 0.37 | 12.49% |
| Chiang 2022. Shanghai (56) 0/1-hour algorithm for Rapid Triage of AMI | Lab/Diagnostic Tests | 1.00 | 0.31 | 0.31 | 12.85% |
| Moore 2007. Moderate to large pelvic fluid ectopic preg. operative in Predicting Need for Operative Intervention | Signs/Symptoms/History | 0.43 | 0.93 | 0.37 | 13.19% |
| Boldrey 1983. Vitreous Pigment in the Diagnosis of Acute PVD | Signs/Symptoms/History | 0.79 | 0.68 | 0.47 | 13.92% |
| Moore 2007. Any fluid in pelvis ectopic preg. operative in Predicting Need for Operative Intervention | Signs/Symptoms/History | 0.72 | 0.73 | 0.46 | 15.06% |
| Bentzer 2016. Hemodynamically unstable, Change in pulse pressure towards Response to Bolus of Intravenous Fluids | Signs/Symptoms/History | 0.62 | 0.83 | 0.45 | 15.70% |
| Herbst 2014. Ultrasound for Diagnosis of Nephrolithiasis | Imaging | 0.73 | 0.73 | 0.46 | 15.80% |
| Martindale 2016. Cephalization in Diagnosis of AHF | Signs/Symptoms/History | 0.45 | 0.95 | 0.39 | 16.19% |
| Runchey 2010. Level of consciousness: alert in Diagnosis of Hemorrhagic Stroke | Signs/Symptoms/History | 0.23 | 0.31 | -0.47 | 16.23% |
| Martindale 2016. Elecsys Roche diagnostic 1550 in Diagnosis of AHF | Lab/Diagnostic Tests | 0.76 | 0.73 | 0.48 | 17.33% |
| Martindale 2016. BIVA in Diagnosis of AHF | Lab/Diagnostic Tests | 0.69 | 0.79 | 0.48 | 17.46% |
| Martindale 2016. BIVA HI in Diagnosis of AHF | Lab/Diagnostic Tests | 0.82 | 0.66 | 0.48 | 17.58% |
| Martindale 2016. Increased LV end diastolic dimension in Diagnosis of AHF | Imaging | 0.80 | 0.69 | 0.48 | 17.72% |
| Fanaroff 2015. Recent episode of similar pain in Diagnosis of ACS | Signs/Symptoms/History | 0.55 | 0.56 | 0.11 | 17.73% |
| Chiang 2022. Tokyo and Taipei (50) 0/1-hour algorithm for Rapid Triage of AMI | Lab/Diagnostic Tests | 1.00 | 0.46 | 0.46 | 18.03% |
| Martindale 2016. Dimension Dade Behring 300 in Diagnosis of AHF | Lab/Diagnostic Tests | 0.96 | 0.48 | 0.44 | 18.16% |
| Chiang 2022. Christchurch (43) 0/2-hour algorithm for Rapid Triage of AMI | Lab/Diagnostic Tests | 0.99 | 0.45 | 0.44 | 18.48% |
| Martindale 2016. Pulmonary edema in Diagnosis of AHF | Signs/Symptoms/History | 0.57 | 0.89 | 0.46 | 18.50% |
| Le 2009. Ocular Ultrasound in Diagnosis of Raised ICP | Imaging | 0.96 | 0.48 | 0.43 | 18.70% |
| Moore 2007. Fluid in right upper quadrant, any ectopic preg. in Predicting Need for Operative Intervention | Signs/Symptoms/History | 0.30 | 1.00 | 0.30 | 18.77% |
| Moore 2007. Moderate to large pelvic fluid any ectopic preg. in Predicting Need for Operative Intervention | Signs/Symptoms/History | 0.48 | 0.95 | 0.43 | 18.98% |
| Chiang 2022. BACC (49) 0/1-hour algorithm for Rapid Triage of AMI | Lab/Diagnostic Tests | 0.99 | 0.47 | 0.47 | 19.56% |
| Sharma 1999. Vitreous Hemorrhage in the Diagnosis of Acute PVD | Signs/Symptoms/History | 0.63 | 0.88 | 0.51 | 19.71% |
| Chiang 2022. Bangkok (54) 0/1-hour algorithm for Rapid Triage of AMI | Lab/Diagnostic Tests | 1.00 | 0.48 | 0.48 | 20.07% |
| Tasman 1968. Vitreous Hemorrhage In Diagnosis of Acute PVD | Signs/Symptoms/History | 0.54 | 0.94 | 0.48 | 21.38% |
| Chiang 2022. HIGH-US (21) 0/1-hour algorithm for Rapid Triage of AMI | Lab/Diagnostic Tests | 0.99 | 0.56 | 0.55 | 21.89% |
| Chiang 2022. APACE (20) 0/3-hour algorithm for Rapid Triage of AMI | Lab/Diagnostic Tests | 0.93 | 0.64 | 0.57 | 22.11% |
| Moak 2012. Ultrasound for Diagnosis of Nephrolithiasis | Imaging | 0.76 | 0.78 | 0.55 | 22.33% |
| Moore 2007. Fluid in pelvis ectopic preg. operative in Predicting Need for Operative Intervention | Signs/Symptoms/History | 0.57 | 0.94 | 0.51 | 22.97% |
| Chiang 2022. APACE (9) 0/3-hour algorithm for Rapid Triage of AMI | Lab/Diagnostic Tests | 0.99 | 0.53 | 0.53 | 23.14% |
| Shah 2012. Chest Radiographs in the Diagnosis of Pneumonia | Imaging | 0.18 | 0.22 | -0.61 | 24.09% |
| Han 2001. Point of Care Ocular Ultrasound for Diagnosis of Retinal Detatchment | Imaging | 0.79 | 0.78 | 0.56 | 24.21% |
| Moore 2007. Any fluid in pelvis any ectopic preg. in Predicting Need for Operative Intervention | Signs/Symptoms/History | 0.54 | 0.74 | 0.28 | 24.59% |
| Martindale 2016. Whole body BIA 292 in Diagnosis of AHF | Lab/Diagnostic Tests | 0.65 | 0.90 | 0.55 | 24.69% |
| Chiang 2022. APACE (20) 0/1-hour algorithm for Rapid Triage of AMI | Lab/Diagnostic Tests | 0.99 | 0.55 | 0.55 | 24.72% |
| Chiang 2022. RAPID-CPU (48) 0/1-hour algorithm for Rapid Triage of AMI | Lab/Diagnostic Tests | 0.99 | 0.56 | 0.55 | 25.44% |
| Chiang 2022. APACE (38) 0/1-hour algorithm for Rapid Triage of AMI | Lab/Diagnostic Tests | 0.99 | 0.59 | 0.58 | 25.68% |
| Chiang 2022. FAST-Ml (52) 0/1-hour algorithm for Rapid Triage of AMI | Lab/Diagnostic Tests | 1.00 | 0.48 | 0.48 | 25.90% |
| Bourcier 2014. Chest CT in the Diagnosis of CAP | Imaging | 0.95 | 0.57 | 0.52 | 26.65% |
| Bourcier 2014. Lung Ultrasound in Diagnosis of CAP | Imaging | 0.95 | 0.57 | 0.52 | 26.65% |
| Martindale 2016. Triage Biosite 200 in Diagnosis of AHF | Lab/Diagnostic Tests | 0.86 | 0.72 | 0.58 | 26.68% |
| Kline 2008. Proximal CUS in Diagnosis of DVT | Imaging | 0.70 | 0.89 | 0.59 | 26.91% |
| Chiang 2022. HIGH-US (21) 0/2-hour algorithm for Rapid Triage of AMI | Lab/Diagnostic Tests | 0.99 | 0.63 | 0.62 | 27.08% |
| Chiang 2022. RAPID-TnT (47) 0/1-hour algorithm for Rapid Triage of AMI | Lab/Diagnostic Tests | 0.97 | 0.75 | 0.72 | 27.12% |
| Rowland 2001. Ultrasound in Diagnosis of Cholelithiasis | Imaging | 0.75 | 0.84 | 0.59 | 27.42% |
| Martindale 2016. Triage Biosite 500 in Diagnosis of AHF | Lab/Diagnostic Tests | 0.68 | 0.90 | 0.57 | 27.46% |
| Chiang 2022. RING (12, 57) 0/3-hour algorithm for Rapid Triage of AMI | Lab/Diagnostic Tests | 0.75 | 0.90 | 0.65 | 28.31% |
| Chiang 2022. HighSTEACS (11) 0/3-hour algorithm for Rapid Triage of AMI | Lab/Diagnostic Tests | 0.91 | 0.74 | 0.65 | 28.42% |
| Hikichi and Trempe 1994. Vitreous Hemorrhage In Diagnosis of Acute PVD | Signs/Symptoms/History | 0.50 | 0.98 | 0.48 | 28.63% |
| Martindale 2016. Reduced EF in Diagnosis of AHF | Imaging | 0.81 | 0.81 | 0.61 | 28.86% |
| Chiang 2022. Lund (46) 0/1-hour algorithm for Rapid Triage of AMI | Lab/Diagnostic Tests | 0.97 | 0.71 | 0.69 | 28.92% |
| Bergstein 1992. Ultrasound and Penetrating Extremity Trauma in Diagnosis of Arterial Injury | Imaging | 0.50 | 0.99 | 0.49 | 29.25% |
| Boldrey 1983. Vitreous Hemorrhage In Diagnosis of Acute PVD | Signs/Symptoms/History | 0.47 | 0.99 | 0.46 | 29.65% |
| Watkins 2007. Ultrasound for Diagnosis of Nephrolithiasis | Imaging | 0.79 | 0.83 | 0.63 | 29.65% |
| Chiang 2022. RING (12,57) 0/2-hour algorithm for Rapid Triage of AMI | Lab/Diagnostic Tests | 1.00 | 0.69 | 0.69 | 29.85% |
| Martindale 2016. iSTAT Abbott 100 in Diagnosis of AHF | Lab/Diagnostic Tests | 0.94 | 0.65 | 0.59 | 30.12% |
| Chiang 2022. APACE (9) 0/3-hour algorithm for Rapid Triage of AMI | Lab/Diagnostic Tests | 0.99 | 0.65 | 0.64 | 30.25% |
| Martindale 2016. Triage Biosite 100 in Diagnosis of AHF | Lab/Diagnostic Tests | 0.93 | 0.53 | 0.46 | 30.26% |
| Turnbull 1990. Color-flow duplex ultrasound in Diagnosis of DVT | Imaging | 0.85 | 0.79 | 0.64 | 30.49% |
| Chiang 2022. RAPID-CPU (48) 0/2-hour algorithm for Rapid Triage of AMI | Lab/Diagnostic Tests | 1.00 | 0.61 | 0.61 | 31.16% |
| Bentzer 2016. Hemodynamically unstable, Controlled ventilation in Response to Bolus of Intravenous Fluids | Signs/Symptoms/History | 0.79 | 0.84 | 0.63 | 31.21% |
| Martindale 2016. AxSym Abbott 100 in Diagnosis of AHF | Lab/Diagnostic Tests | 0.93 | 0.53 | 0.46 | 31.22% |
| Chiang 2022. APACE (20) 0/2-hour algorithm for Rapid Triage of AMI | Lab/Diagnostic Tests | 0.99 | 0.67 | 0.65 | 31.98% |
| Corradi 2015. Lung Ultrasound in Diagnosis of CAP | Imaging | 0.68 | 0.95 | 0.63 | 32.30% |
| Runchey 2010. Hemorrhage most likely diagnosis in Diagnosis of Hemorrhagic Stroke | Signs/Symptoms/History | 0.76 | 0.88 | 0.64 | 32.31% |
| Bentzer 2016. Hemodynamically unstable, Controlled ventilation V <7.0 mL/kg in Response to Bolus of Intravenous Fluids | Signs/Symptoms/History | 0.72 | 0.91 | 0.63 | 32.48% |
| Chiang 2022. OUT-ACS (51) 0/1-hour algorithm for Rapid Triage of AMI | Lab/Diagnostic Tests | 0.98 | 0.79 | 0.78 | 32.67% |
| Chiang 2022. Brighton and Sussex (53) 0/1-hour algorithm for Rapid Triage of AMI | Lab/Diagnostic Tests | 1.00 | 0.72 | 0.72 | 34.57% |
| Davis 2005. Ultrasound in Diagnosis of Cholelithiasis | Imaging | 0.81 | 0.86 | 0.67 | 34.77% |
| Iverson 2012. Point of Care Ultrasonography for the Diagnosis of Abscess | Imaging | 0.97 | 0.67 | 0.63 | 35.34% |
| Moore 2007. Fluid in right upper quadrant, ectopic preg. operative in Predicting Need for Operative Intervention | Signs/Symptoms/History | 0.50 | 1.00 | 0.50 | 35.35% |
| Chiang 2022. Christchurch (41) 0/1-hour algorithm for Rapid Triage of AMI | Lab/Diagnostic Tests | 1.00 | 0.68 | 0.68 | 35.43% |
| Mantuani 2016. Lung Ultrasound in Diagnosis of CAP | Imaging | 0.84 | 0.84 | 0.68 | 36.25% |
| Bentzer 2016. Hemodynamically unstable, Controlled ventilation V ≥7.0 mL/kg in Response to Bolus of Intravenous Fluids | Signs/Symptoms/History | 0.84 | 0.84 | 0.68 | 36.84% |
| Chiang 2022. Brisbane (35) 0/2-hour algorithm for Rapid Triage of AMI | Lab/Diagnostic Tests | 0.91 | 0.85 | 0.76 | 37.50% |
| Gaspari 2005. Ultrasound for Diagnosis of Nephrolithiasis | Imaging | 0.87 | 0.82 | 0.69 | 38.11% |
| Martindale 2016. Elecsys Roche diagnostic 1000 in Diagnosis of AHF | Lab/Diagnostic Tests | 0.85 | 0.65 | 0.50 | 38.11% |
| Chu 2017. Point of Care Ocular Ultrasound for Diagnosis of Retinal Detatchment | Imaging | 0.88 | 0.87 | 0.74 | 38.13% |
| Chiang 2022. RING (12, 57) 0/3-hour algorithm for Rapid Triage of AMI | Lab/Diagnostic Tests | 0.75 | 0.95 | 0.70 | 38.44% |
| Chiang 2022. Brisbane (35) 0/2-hour algorithm for Rapid Triage of AMI | Lab/Diagnostic Tests | 0.96 | 0.83 | 0.79 | 38.87% |
| Kim 2018. Point of Care Ocular Ultrasound for Diagnosis of Retinal Detatchment | Imaging | 0.75 | 0.94 | 0.69 | 39.37% |
| Chiang 2022. HighSTEACS (10) 0/1-hour algorithm for Rapid Triage of AMI | Lab/Diagnostic Tests | 1.00 | 0.78 | 0.78 | 39.48% |
| Fanaroff 2015. Associated dyspnea in Diagnosis of ACS | Signs/Symptoms/History | 0.45 | 0.61 | 0.06 | 39.67% |
| Runchey 2010. Xanthochromia in cerebrospinal fluid in Diagnosis of Hemorrhagic Stroke | Lab/Diagnostic Tests | 0.71 | 0.95 | 0.67 | 39.78% |
| Berger 2012. Point of Care Ultrasonography for the Diagnosis of Abscess | Imaging | 0.97 | 0.67 | 0.64 | 39.86% |
| Sivitz 2010. Point of Care Ultrasonography for the Diagnosis of Abscess | Imaging | 0.91 | 0.83 | 0.74 | 40.67% |
| Sharma 1999. Vitreous Pigment or Vitreous Hemorrhage in Diagnosis of Acute PVD | Signs/Symptoms/History | 0.88 | 0.88 | 0.76 | 41.11% |
| Rosen 2001. Ultrasound in Diagnosis of Cholelithiasis | Imaging | 0.92 | 0.78 | 0.70 | 41.17% |
| Pagano 2015. Lung Ultrasound in Diagnosis of CAP | Imaging | 0.99 | 0.65 | 0.63 | 41.73% |
| Martindale 2016. Restrictive mitral pattern in Diagnosis of AHF | Imaging | 0.81 | 0.90 | 0.72 | 41.86% |
| Chiang 2022. BACC (49) 0/3-hour algorithm for Rapid Triage of AMI | Lab/Diagnostic Tests | 0.83 | 0.91 | 0.74 | 41.91% |
| Frazee 2001. Proximal CUS in Diagnosis of DVT | Imaging | 0.78 | 0.93 | 0.71 | 42.10% |
| Shah 2013. Lung Ultrasound in Diagnosis of Pneumonia in Childhood | Imaging | 0.86 | 0.89 | 0.75 | 42.54% |
| Chiang 2022. RAPID-CPU (48) 0/3-hour algorithm for Rapid Triage of AMI | Lab/Diagnostic Tests | 0.97 | 0.77 | 0.74 | 42.84% |
| Chiang 2022. HighSTEACS (10) 0/3-hour algorithm for Rapid Triage of AMI | Lab/Diagnostic Tests | 0.93 | 0.84 | 0.77 | 43.49% |
| Fanaroff 2015. Radiation to left arm in Diagnosis of ACS | Signs/Symptoms/History | 0.40 | 0.69 | 0.09 | 44.19% |
| Martindale 2016. Absent Fever in Diagnosis of AHF | Signs/Symptoms/History | 0.92 | 0.21 | 0.13 | 46.21% |
| Inaba 2011. MDCT and Penetrating Extremity Trauma in Diagnosis of Arterial Injury (Performance of Hard Signs) | Imaging | 0.59 | 1.00 | 0.59 | 46.29% |
| Bentzer 2016. Hemodynamically unstable, Spontaneous breathing in Response to Bolus of Intravenous Fluids | Signs/Symptoms/History | 0.88 | 0.88 | 0.76 | 46.81% |
| Chiang 2022. HighSTEACS (11) 0/1-hour algorithm for Rapid Triage of AMI | Lab/Diagnostic Tests | 0.94 | 0.69 | 0.63 | 46.85% |
| Chiang 2022. RAPIO-TnT (47) 0/3-hour algorithm for Rapid Triage of AMI | Lab/Diagnostic Tests | 0.88 | 0.95 | 0.82 | 47.33% |
| Novak and Welch 1984. Vitreous Hemorrhage In Diagnosis of Acute PVD | Signs/Symptoms/History | 0.79 | 0.96 | 0.75 | 48.79% |
| Bentzer 2016. Hemodynamically unstable, Controlled ventilation in Response to Bolus of Intravenous Fluids | Signs/Symptoms/History | 0.77 | 0.85 | 0.62 | 49.67% |
| Martindale 2016. Positive B-line scan in Diagnosis of AHF | Imaging | 0.85 | 0.93 | 0.78 | 50.75% |
| Schwartz 1993. Ultrasound and Penetrating Extremity Trauma in Diagnosis of Arterial Injury (Performance of Ankle-Brachial Index) | Imaging | 0.47 | 0.85 | 0.32 | 51.44% |
| Chiang 2022. RING (12, 57) 0/2-hour algorithm for Rapid Triage of AMI | Lab/Diagnostic Tests | 1.00 | 0.66 | 0.66 | 52.01% |
| Testa 2012. CXR, Chest CT, & Suspected H1N1 Infection in Diagnosis of Pneumonia | Imaging | 0.94 | 0.85 | 0.79 | 52.62% |
| Martindale 2016. Segmental BIA in Diagnosis of AHF | Lab/Diagnostic Tests | 0.88 | 0.92 | 0.80 | 52.79% |
| Nazerian 2015. Chest CT & Respiratory Complaint in the Diagnosis of Pneumonia | Imaging | 0.83 | 0.95 | 0.78 | 53.33% |
| Nazerian 2015. Lung Ultrasound in Diagnosis of CAP | Imaging | 0.83 | 0.95 | 0.78 | 53.33% |
| Martindale 2016. Kerley B-lines in Diagnosis of AHF | Imaging | 0.09 | 0.99 | 0.08 | 54.10% |
| Unluer 2013. CXR + Chest CT in the Diagnosis of Pneumonia | Imaging | 0.96 | 0.84 | 0.81 | 54.46% |
| Alexander 2008. Ultrasound in Diagnosis of Cholelithiasis | Imaging | 0.86 | 0.95 | 0.81 | 55.71% |
| Chiang 2022. Christchurch (12) 0/3-hour algorithm for Rapid Triage of AMI | Lab/Diagnostic Tests | 0.79 | 0.98 | 0.77 | 55.77% |
| Schwartz 1993. Ultrasound and Penetrating Extremity Trauma in Diagnosis of Arterial Injury (Performance of Any Sign in Combination) | Imaging | 1.00 | 0.54 | 0.54 | 55.83% |
| Chiang 2022. Christchurch (12) 0/3-hour algorithm for Rapid Triage of AMI | Lab/Diagnostic Tests | 0.70 | 0.99 | 0.70 | 56.37% |
| Karakitsos 2006. Ocular Ultrasound in Diagnosis of Raised ICP | Imaging | 0.67 | 0.94 | 0.61 | 56.98% |
| Samson 2016. Lung Ultrasound in Diagnosis of Pneumonia in Childhood | Imaging | 0.87 | 0.95 | 0.82 | 57.41% |
| Adams 2016. Point of Care Ultrasonography for the Diagnosis of Abscess | Imaging | 0.96 | 0.88 | 0.84 | 58.24% |
| Tayal 2007. Ocular Ultrasound in Diagnosis of Raised ICP | Imaging | 1.00 | 0.63 | 0.63 | 58.44% |
| Inaba 2011. MDCT and Penetrating Extremity Trauma in Diagnosis of Arterial Injury (Performance of Any Sign in Combination) | Imaging | 1.00 | 0.87 | 0.87 | 58.82% |
| Bentzer 2016. Hemodynamically unstable, Controlled ventilation in Response to Bolus of Intravenous Fluids | Signs/Symptoms/History | 0.92 | 0.92 | 0.84 | 59.21% |
| Lichtenstein 2008. CXR, Chest CT, and Acute Respiratory Failure in the Diagnosis of Pneumonia | Imaging | 0.89 | 0.94 | 0.84 | 59.35% |
| Chiang 2022. BACC (38) 0/1-hour algorithm for Rapid Triage of AMI | Lab/Diagnostic Tests | 0.99 | 0.61 | 0.60 | 59.95% |
| Gonzalez 1999. Physical Examination and Penetrating Extremity Trauma in Diagnosis of Arterial Injury | Signs/Symptoms/History | 0.92 | 0.95 | 0.87 | 60.17% |
| Kendall 2001. Ultrasound in Diagnosis of Cholelithiasis | Imaging | 0.96 | 0.88 | 0.84 | 60.69% |
| Boursiani 2017. Lung Ultrasound in Diagnosis of Pneumonia in Childhood | Imaging | 0.94 | 1.00 | 0.94 | 61.26% |
| Zamani Moghadam 2017. Ultrasound of Confirmation of Endotracheal Tube Intubation | Imaging | 0.97 | 0.88 | 0.85 | 62.03% |
| Yoonessi 2010. Point of Care Ocular Ultrasound for Diagnosis of Retinal Detatchment | Imaging | 1.00 | 0.83 | 0.83 | 62.08% |
| Yoonessi 2010. Ocular Ultrasound for Diagnosis of Retinal Detachment | Imaging | 1.00 | 0.83 | 0.83 | 62.08% |
| Jacoby 2007. Proximal CUS in Diagnosis of DVT | Imaging | 0.89 | 0.97 | 0.86 | 62.11% |
| Saglam 2012. Ultrasound of Confirmation of Endotracheal Tube Intubation | Imaging | 0.97 | 0.80 | 0.77 | 62.33% |
| Chiang 2022. Calgary (19) 0/2-hour algorithm for Rapid Triage of AMI | Lab/Diagnostic Tests | 1.00 | 0.72 | 0.72 | 62.82% |
| Dean 2004. Proximal CUS in Diagnosis of DVT | Imaging | 0.88 | 0.97 | 0.85 | 63.71% |
| Summers 2010. Ultrasound in Diagnosis of Cholelithiasis | Imaging | 0.89 | 0.86 | 0.75 | 63.87% |
| Chiang 2022. Christchurch (42–45) 0/2-hour algorithm for Rapid Triage of AMI | Lab/Diagnostic Tests | 0.98 | 0.71 | 0.69 | 64.22% |
| Taylor 2010. Hepatomegaly in Diagnosis of Malaria | Signs/Symptoms/History | 0.10 | 0.97 | 0.07 | 64.96% |
| Inaba 2011. MDCT and Penetrating Extremity Trauma in Diagnosis of Arterial Injury (Performance of ABI) | Imaging | 0.56 | 0.95 | 0.51 | 65.31% |
| Squire 2005. Point of Care Ultrasonography for the Diagnosis of Abscess | Imaging | 0.98 | 0.89 | 0.87 | 65.77% |
| Chiang 2022. APACE (38) 0/1-hour algorithm for Rapid Triage of AMI | Lab/Diagnostic Tests | 0.99 | 0.73 | 0.72 | 65.83% |
| Shinar 2011. Point of Care Ocular Ultrasound for Diagnosis of Retinal Detatchment | Imaging | 0.97 | 0.92 | 0.89 | 66.68% |
| Shinar 2011. Ocular Ultrasound for Diagnosis of Retinal Detachment | Imaging | 0.97 | 0.92 | 0.89 | 66.68% |
| Helmke 1996. Ocular Ultrasound in Diagnosis of Raised ICP | Imaging | 1.00 | 0.86 | 0.86 | 66.83% |
| Bentzer 2016. Hemodynamically unstable, Change in cardiac output towards Response to Bolus of Intravenous Fluids | Signs/Symptoms/History | 0.88 | 0.92 | 0.80 | 67.69% |
| Miller 2006. Ultrasound in Diagnosis of Cholelithiasis | Imaging | 0.94 | 0.96 | 0.90 | 67.70% |
| Chiang 2022. Christchurch (41) 0/1-hour algorithm for Rapid Triage of AMI | Lab/Diagnostic Tests | 0.97 | 0.78 | 0.75 | 68.15% |
| Reali 2014. Lung Ultrasound in Diagnosis of Pneumonia in Childhood | Imaging | 0.94 | 0.96 | 0.90 | 68.22% |
| Ellington 2017. Lung Ultrasound in Diagnosis of Pneumonia in Childhood | Imaging | 0.90 | 0.97 | 0.88 | 68.39% |
| Gallard 2015. Lung Ultrasound in Diagnosis of CAP | Imaging | 0.74 | 0.94 | 0.68 | 68.68% |
| Claes 2016. Lung Ultrasound in Diagnosis of Pneumonia in Childhood | Imaging | 0.98 | 0.92 | 0.90 | 68.75% |
| Ha 2002. Ultrasound in Diagnosis of Cholelithiasis | Imaging | 0.94 | 0.95 | 0.89 | 69.01% |
| Lichtenstein 2004. Chest CT & Chest Pain or Severe Thoracic Diseases in the Diagnosis of Pneumonia | Imaging | 0.91 | 0.98 | 0.89 | 69.81% |
| Tabotabo 1980. Vitreous Hemorrhage In Diagnosis of Acute PVD | Signs/Symptoms/History | 1.00 | 0.93 | 0.93 | 69.83% |
| Quraishi 1997. Point of Care Ultrasonography for the Diagnosis of Abscess | Imaging | 0.65 | 1.00 | 0.65 | 69.87% |
| Rice 2009. Proximal CUS in Diagnosis of DVT | Imaging | 0.90 | 0.93 | 0.82 | 70.38% |
| Anderson 1990. Arteriography and Penetrating Extremity Trauma in Diagnosis of Arterial Injury | Signs/Symptoms/History | 0.83 | 1.00 | 0.83 | 70.74% |
| Chance 1991. Proximal CUS in Diagnosis of DVT | Imaging | 1.00 | 0.93 | 0.93 | 72.78% |
| Knaut 2005. Ultrasound in Diagnosis of Suspected AAA | Imaging | 1.00 | 0.97 | 0.97 | 73.61% |
| Jaffe 1968. Vitreous Hemorrhage In Diagnosis of Acute PVD | Signs/Symptoms/History | 1.00 | 0.95 | 0.95 | 74.00% |
| Dimitrios 2017. Lung Ultrasound in Diagnosis of CAP | Imaging | 1.00 | 0.97 | 0.97 | 74.27% |
| Jang 2004. Proximal CUS in Diagnosis of DVT | Imaging | 1.00 | 0.92 | 0.92 | 74.89% |
| Abhishek 2017. Ultrasound of Confirmation of Endotracheal Tube Intubation | Imaging | 0.97 | 1.00 | 0.97 | 75.47% |
| Lanoix 2000. Ultrasound in Diagnosis of Suspected AAA | Imaging | 1.00 | 0.94 | 0.94 | 75.53% |
| Yadav 2017. Lung Ultrasound in Diagnosis of Pneumonia in Childhood | Imaging | 0.98 | 0.65 | 0.63 | 76.55% |
| Esposito 2014. CR in the Diagnosis of CAP | Imaging | 0.98 | 0.94 | 0.92 | 76.87% |
| Goel 2008. Ocular Ultrasound in Diagnosis of Raised ICP | Imaging | 0.99 | 0.93 | 0.91 | 76.97% |
| Ünlüer 2013. Lung Ultrasound in Diagnosis of CAP | Imaging | 0.96 | 0.84 | 0.81 | 77.45% |
| Chou 2013. Ultrasound of Confirmation of Endotracheal Tube Intubation | Imaging | 1.00 | 0.86 | 0.86 | 77.92% |
| Sun 2014. Ultrasound of Confirmation of Endotracheal Tube Intubation | Imaging | 1.00 | 0.86 | 0.86 | 78.08% |
| Reissig 2012. CXR & Chest CT if CXR/LUS discordance in the Diagnosis of CAP | Imaging | 0.93 | 0.98 | 0.91 | 78.23% |
| Nafae 2013. Chest CT in the Diagnosis of Pneumonia | Imaging | 0.98 | 0.94 | 0.93 | 78.94% |
| Thomas 2017. Ultrasound of Confirmation of Endotracheal Tube Intubation | Imaging | 0.98 | 1.00 | 0.98 | 80.38% |
| Cortellaro 2010. CXR & Chest CT in the Diagnosis of CAP | Imaging | 0.99 | 0.95 | 0.94 | 81.46% |
| Cortellaro 2012. Lung Ultrasound in Diagnosis of CAP | Imaging | 0.99 | 0.95 | 0.94 | 81.46% |
| Yang 2017. Ultrasound of Confirmation of Endotracheal Tube Intubation | Imaging | 1.00 | 0.89 | 0.89 | 81.61% |
| Chiang 2022. Brighton and Sussex (53) 0/3-hour algorithm for Rapid Triage of AMI | Lab/Diagnostic Tests | 0.95 | 0.99 | 0.94 | 81.62% |
| Fojtik 2003. Proximal CUS in Diagnosis of DVT | Imaging | 0.96 | 0.99 | 0.94 | 82.18% |
| Lahham 2017. Ultrasound of Confirmation of Endotracheal Tube Intubation | Imaging | 0.99 | 1.00 | 0.99 | 82.97% |
| Laursen 2014. Lung Ultrasound in Diagnosis of CAP | Imaging | 0.93 | 1.00 | 0.93 | 83.42% |
| Liu 2015. Lung Ultrasound in Diagnosis of CAP | Imaging | 0.94 | 1.00 | 0.94 | 83.99% |
| Kuhn 2000. Ultrasound in Diagnosis of Suspected AAA | Imaging | 1.00 | 0.95 | 0.95 | 84.07% |
| Kuzniec 1998. Ultrasound and Penetrating Extremity Trauma in Diagnosis of Arterial Injury | Imaging | 0.94 | 1.00 | 0.94 | 84.25% |
| Blaivas 2003. Ocular Ultrasound in Diagnosis of Raised ICP | Imaging | 1.00 | 0.95 | 0.95 | 84.40% |
| Milling 2007. Ultrasound of Confirmation of Endotracheal Tube Intubation | Imaging | 0.97 | 1.00 | 0.97 | 84.75% |
| Iorio 2015. Lung Ultrasound in Diagnosis of Pneumonia in Childhood | Imaging | 0.97 | 0.96 | 0.92 | 85.72% |
| Beare 2008. Ocular Ultrasound in Diagnosis of Raised ICP | Imaging | 0.93 | 1.00 | 0.93 | 86.04% |
| Skoloudik 2011. Ocular Ultrasound in Diagnosis of Raised ICP | Imaging | 0.81 | 1.00 | 0.81 | 86.12% |
| Chou 2011. Ultrasound of Confirmation of Endotracheal Tube Intubation | Imaging | 0.99 | 0.94 | 0.93 | 86.33% |
| Reissig 2012. Lung Ultrasound in Diagnosis of CAP | Imaging | 0.93 | 0.98 | 0.91 | 87.18% |
| Masoumi 2017. Ultrasound of Confirmation of Endotracheal Tube Intubation | Imaging | 0.99 | 1.00 | 0.99 | 87.35% |
| Kongsap 2011. Point of Care Ocular Ultrasound for Diagnosis of Retinal Detatchment | Imaging | 1.00 | 0.99 | 0.99 | 87.94% |
| Tayal 2003. Ultrasound in Diagnosis of Suspected AAA | Imaging | 1.00 | 0.98 | 0.98 | 88.84% |
| Espocito 2014. Lung Ultrasound in Diagnosis of Pneumonia in Childhood | Imaging | 0.98 | 0.95 | 0.92 | 88.94% |
| Magazzini 2007. Complete CUS in Diagnosis of DVT | Imaging | 1.00 | 0.98 | 0.98 | 90.18% |
| Theodoro 2004. Proximal CUS in Diagnosis of DVT | Imaging | 1.00 | 0.98 | 0.98 | 90.39% |
| McNicholas 1995. Point of Care Ocular Ultrasound for Diagnosis of Retinal Detatchment | Imaging | 0.81 | 1.00 | 0.81 | 90.49% |
| Caiulo 2012. Lung Ultrasound in Diagnosis of Pneumonia in Childhood | Imaging | 0.99 | 1.00 | 0.99 | 90.74% |
| Qayyum 2012. Ocular Ultrasound in Diagnosis of Raised ICP | Imaging | 1.00 | 0.75 | 0.75 | 90.98% |
| Xirouchaki 2011. Chest CT & Mechanical Ventilation in the Diagnosis of Pneumonia | Imaging | 1.00 | 0.78 | 0.78 | 91.10% |
| Anderson 1990. Arteriography and Penetrating Extremity Trauma in Diagnosis of Arterial Injury (Performance of ABI) | Signs/Symptoms/History | 0.67 | 1.00 | 0.67 | 91.58% |
| Parlamento 2009. CXR & Chest CT if CXR/LUS discordance in the Diagnosis of CAP | Imaging | 0.97 | 1.00 | 0.97 | 92.28% |
| Parlamento 2009. Lung Ultrasound in Diagnosis of CAP | Imaging | 0.97 | 1.00 | 0.97 | 92.29% |
| Park 2009. Ultrasound of Confirmation of Endotracheal Tube Intubation | Imaging | 0.96 | 1.00 | 0.96 | 92.33% |
| Abbasi 2015. Ultrasound of Confirmation of Endotracheal Tube Intubation | Imaging | 0.99 | 1.00 | 0.99 | 92.56% |
| Urbankowska 2015. Lung Ultrasound in Diagnosis of Pneumonia in Childhood | Imaging | 0.93 | 1.00 | 0.93 | 92.68% |
| Adi 2013. Ultrasound of Confirmation of Endotracheal Tube Intubation | Imaging | 0.98 | 1.00 | 0.98 | 93.09% |
| Jones 2003. Ultrasound in Diagnosis of Suspected AAA | Imaging | 0.98 | 1.00 | 0.98 | 94.29% |
| Tayal 2006. Point of Care Ultrasonography for the Diagnosis of Abscess | Imaging | 1.00 | 0.93 | 0.93 | 94.48% |
| Liu 2014. CT Scan in the Diagnosis of CAP | Imaging | 0.94 | 1.00 | 0.94 | 94.70% |
| Lichtenstein 2004. Chest CT & Acute Respiratory Distress Syndrome in the Diagnosis of Pneumonia | Imaging | 0.93 | 1.00 | 0.93 | 95.61% |
| Sharma 1999. Vitreous Pigment in Diagnosis of Acute PVD | Signs/Symptoms/History | 0.63 | 1.00 | 0.63 | 95.81% |
| Interrigi 2017. Lung Ultrasound in Diagnosis of CAP | Imaging | 0.86 | 1.00 | 0.86 | 96.01% |
| Sharma 1999. Vitreous Pigment and Vitreous Hemorrhage in Diagnosis of Acute PVD | Signs/Symptoms/History | 0.38 | 1.00 | 0.38 | 96.34% |
| Zanobetti 2017. Lung Ultrasound in Diagnosis of CAP | Imaging | 1.00 | 0.99 | 0.99 | 96.86% |
| Linder 1966. Vitreous Hemorrhage In Diagnosis of Acute PVD | Signs/Symptoms/History | 0.87 | 1.00 | 0.87 | 97.26% |
| Caiulo VA 2013. Chest Radiographs in the Diagnosis of Pneumonia | Imaging | 0.99 | 1.00 | 0.99 | 97.86% |
| Shiver 2010. Proximal CUS in Diagnosis of DVT | Imaging | 0.83 | 1.00 | 0.83 | 97.90% |
| Brod 1991. Vitreous Pigment in Diagnosis of Acute PVD | Signs/Symptoms/History | 0.93 | 1.00 | 0.93 | 98.31% |
| Major 2011. Ocular Ultrasound in Diagnosis of Raised ICP | Imaging | 0.86 | 1.00 | 0.86 | 98.51% |
| Blaivas 2000. Color-flow duplex ultrasound in Diagnosis of DVT | Imaging | 1.00 | 0.99 | 0.99 | 98.57% |
| Crisp 2010. Proximal CUS in Diagnosis of DVT | Imaging | 1.00 | 0.99 | 0.99 | 99.07% |
| Tanner 2000. Vitreous Pigment In Diagnosis of Acute PVD | Signs/Symptoms/History | 0.93 | 1.00 | 0.93 | 99.68% |
| Abbasi 2013. Ultrasonographic Tests for Shoulder Dislocation Detection | Imaging | 1.00 | 1.00 | 1.00 | 100.00% |
| Abbasi 2013. Ultrasonographic Tests for Shoulder Reduction Confirmation | Imaging | 1.00 | 1.00 | 1.00 | 100.00% |
| Benci 1996. CXR & Chest CT if CXR/LUS discordance in the Diagnosis of Pneumonia | Imaging | 1.00 | 1.00 | 1.00 | 100.00% |
| Copetti 2008. ECG, X-Ray, & CD Echo on Acute Pulmonary Edema Patients in Diagnosis of Pneumonia | Imaging | 1.00 | 1.00 | 1.00 | 100.00% |
| Ukponmwan 2001. Point of Care Ocular Ultrasound for Diagnosis of Retinal Detatchment | Imaging | 1.00 | 1.00 | 1.00 | 100.00% |
| Blaivas 2002. Point of Care Ocular Ultrasound for Diagnosis of Retinal Detatchment | Imaging | 1.00 | 1.00 | 1.00 | 100.00% |
| Nagaraju 2015. Point of Care Ocular Ultrasound for Diagnosis of Retinal Detatchment | Imaging | 1.00 | 1.00 | 1.00 | 100.00% |
| Werner 2007. Ultrasound of Confirmation of Endotracheal Tube Intubation | Imaging | 1.00 | 1.00 | 1.00 | 100.00% |
| Muslu 2011. Ultrasound of Confirmation of Endotracheal Tube Intubation | Imaging | 1.00 | 1.00 | 1.00 | 100.00% |
| Hoffman 2014. Ultrasound of Confirmation of Endotracheal Tube Intubation | Imaging | 1.00 | 1.00 | 1.00 | 100.00% |
| Rajajee 2010. Ocular Ultrasound in Diagnosis of Raised ICP | Imaging | 1.00 | 1.00 | 1.00 | 100.00% |
| Liu 2014. Lung Ultrasound in Diagnosis of Pneumonia in Childhood | Imaging | 1.00 | 1.00 | 1.00 | 100.00% |
| Copetti 2008. Lung Ultrasound in Diagnosis of Pneumonia in Childhood | Imaging | 1.00 | 1.00 | 1.00 | 100.00% |
| Farahmand 2011. Proximal CUS in Diagnosis of DVT | Imaging | 1.00 | 1.00 | 1.00 | 100.00% |
| Rowland 2001. Ultrasound in Diagnosis of Suspected AAA | Imaging | 1.00 | 1.00 | 1.00 | 100.00% |
| Costantino 2005. Ultrasound in Diagnosis of Suspected AAA | Imaging | 1.00 | 1.00 | 1.00 | 100.00% |
| Blaivas 2000. Point-of-care Ocular Ultrasound for Diagnosis of Retinal Detachment | Imaging | 1.00 | 1.00 | 1.00 | 100.00% |

**Supplementary Table S1. Full dataset of 405 clinical features** **grouped by category (demographics, signs/symptoms, laboratory tests, imaging).**Each feature is reported with sensitivity, specificity, Youden’s Index, and entropy removal percentage. These values were used to construct the quadrant analysis in Fig. 1 and Tables 1–4.

A.
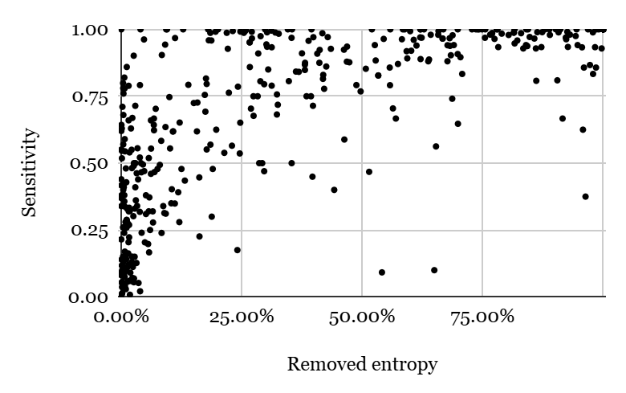
B.
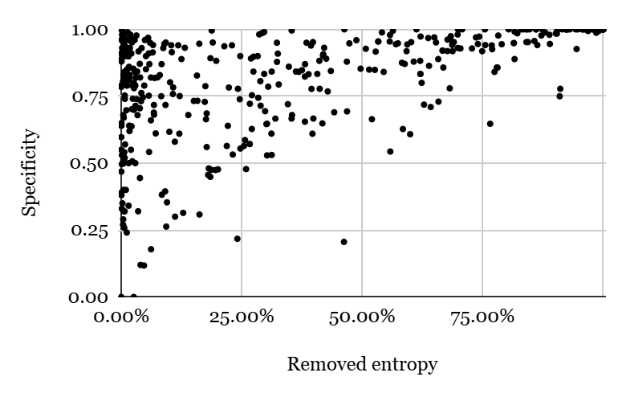

**Supplementary Figure S1. Scatterplots of removed entropy vs. sensitivity or specificity.**
Includes the data of 405 clinical symptoms, demographics, and diagnostic test findings.
(A) Sensitivity expressed a 0.70 Pearson correlation and a 0.76 Spearman correlation with entropy removal (p<0.001).
(B) Specificity expressed a 0.50 Pearson correlation and a 0.56 Spearman correlation with entropy removal (p<0.001).

A.
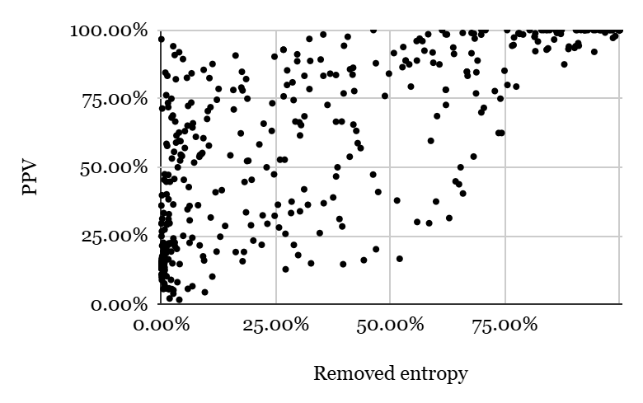
B.
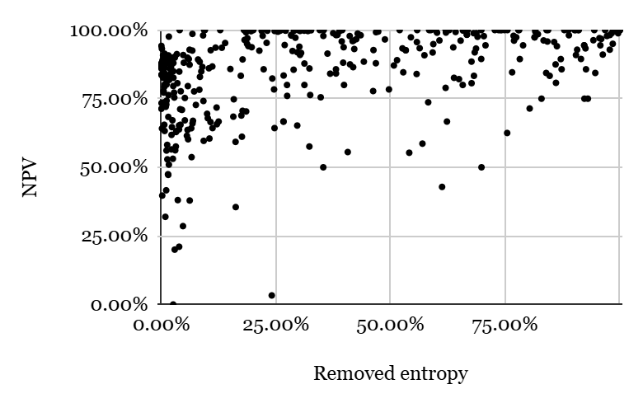

**Supplementary Figure S2. Scatterplots of removed entropy vs. positive predictive value (%) or negative predictive value (%).**
Includes the data of 405 clinical symptoms, demographics, and diagnostic test findings.
(A) PPV expressed a 0.74 Pearson correlation and a 0.78 Spearman correlation with entropy removal (p < 0.001).
(B) NPV expressed a 0.43 Pearson correlation and a 0.53 Spearman correlation with entropy removal (p < 0.001).
